# Supplementary material for: Epigenomic profiling of primate lymphoblastoid cell lines reveals the evolutionary patterns of epigenetic activities in gene regulatory architectures
Source: Nat Commun. 2021 May 25;12:3116. doi: 10.1038/s41467-021-23397-1 (PMC8149829; doi:10.1038/s41467-021-23397-1)
Supplement: Supplementary file 1 — Supplementary Information [file 41467_2021_23397_MOESM1_ESM.pdf]

Supplementary Information

containing Supplementary Methods, Supplementary Figures, Supplementary Tables for

Epigenomic profiling of primate lymphoblastoid cell lines reveals the evolutionary patterns of epigenetic activities in gene regulatory architectures

Raquel García-Pérez[*#], Paula Esteller-Cucala[#], Glòria Mas, Irene Lobón, Valerio di Carlo, Meritxell Riera, Martin Kuhlwilm, Arcadi Navarro, Antoine Blancher, Luciano Di Croce, José Luis Gómez-Skarmeta, David Juan[*#], Tomàs Marquès-Bonet[*#]

This PDF file includes the description and/or content of:

Supplementary Methods

Supplementary Figures 1 to 43

Supplementary Tables 1 to 9

Supplementary Methods

Quality control

Sequence quality was assessed using the FASTQC (version 0.11.4) software[1] for all types of generated sequencing data. Quality filtering and adapter removal were performed when deemed necessary using Cutadapt (version 1.10)[2] to ensure that raw reads were within standard parameters. We constructed species-specific mappability tracks for the different sequencing read lengths included in this study (151-kmers, 101-kmers and 50-kmers) in order to identify positions in the reference genomes where reads could not be confidently mapped. We used the 'gem-mappability' module from gemtools/1.7[3], which uses a kmer approach to detect genomic regions that are duplicated and, therefore, likely to be problematic. For paired-end data, we considered mappable regions those with up to 2 mismatches (threshold ≥ 0.5). For single-end data, we considered mappable regions those with less than 2 mismatches (threshold > 0.5)

ChIP-seq and ATAC-seq short-read alignment and peak calling

ChIP-seq 50 bp SE reads were aligned to the corresponding reference genome along with the genome of the Epstein-Barr virus with Bowtie2 (version 2.2.11)[4] using the default 'sensitive' settings. Low-

quality and multiple-mapping reads were removed using Samtools (version 1.2)[5] with options '-q30 –F 1796' ('-q30 -F 1804 -f 2' for sample GM12878 that was sequenced with 75 bp PE reads). PCR duplicates were removed using Picard (version 1.95) (http://broadinstitute.github.io/picard/). Reads mapping to the corresponding species genome were then selected and only autosomal chromosomes were kept for downstream analysis. We identified enriched regions or peaks with MACS2 (version 2.1.1)[6] using the following parameters *-nomodel --shift 0 –extsize sample_specific_fragment_size --keep-dup all* for histones H3K4me3, H3K4me1 and H3K27ac, and adding *–broad* for histones H3K36me3 and H3K27me3. We only kept reproducible peaks, which we defined following a partition concordance approach. For each sample, we created two pseudo-replicates (defined by randomly choosing half of each sample reads without replacement) and used them to call peaks using the above-stated command. We defined as reproducible peaks those that overlapped at least 50% with peaks from both sample pseudo-replicates. Samples peaks were filtered based on their corresponding 50-kmer mappability tracks and only peaks with at least 80% mappable bp were retained. Histone peaks were strongly enriched in genomic regions with consistent chromatin states (Supplementary Figs. 2 and 33).

ATAc-seq 50 bp PE reads were mapped to the corresponding reference genome along with the genome of the Epstein-Barr virus using Bowtie2 (version 2.2.11)[4] with default 'sensitive' settings and '--maxins 10000' that allows the mapping of reads with a maximum insert size of 10 kbp. Low-quality and multiple-mapping reads were removed using Samtools (version 1.2)[5] with options '-q30 -F 1804 -f 2'. PCR duplicates were removed using Picard (version 1.95) (http://broadinstitute.github.io/picard/). Only autosomal chromosomes were kept for downstream analyses. Open chromatin regions were called using the ENCODE ATAC-seq pipeline (https://encode-dcc.github.io/wdl-pipelines/), with an IDR threshold of 0.1. Open chromatin regions were further filtered based on 50-kmer species-specific mappability tracks and only accessible regions with at least 80% mappable bp were retained. We applied the same approach as before to retain reproducible open chromatin regions. Open chromatin regions were strongly enriched in genomic regions with consistent chromatin states (Supplementary Figs. 2 and 33, Supplementary Data 19).

Background noise normalization of enrichment values

To obtain background noise corrected histone enrichment signals, we implemented a 3-step approach. First, sample matched immunoprecipitated (IP) and input counts were normalized by sequencing depth. The median number of aligned reads across samples IP and input datasets (for any given histone mark) was used as the total reference count. IP and input counts were then scaled to the total reference count. Second, we removed background noise in a sample- and histone modification-specific fashion. The input-IP relationship in genomic regions with no significant enrichment (non-peaks) was used to estimate the noise contribution to the IP in peaks. We defined a confident set of non-peaks with the

same size and same mappability requirements as for the identified samples peaks that did not overlap with any peak detected in any of the five species considered. These strict requirements prevented the inclusion of putative false negatives, enriched regions that were overlooked by the peak calling algorithm. We used a Deming regression to evaluate the linear relationship between the input and IP in non-peaks. Deming regression assumes random measurement error in both the dependent y-variable and the independent x-variable[7], and thus, is more suitable for these analyses than simple linear regression where only the response variable Y is measured with error (Supplementary Fig. 34). The noise contribution to the IP was estimated for each genomic region of interest using the inferred linear input-IP relationship in non-peaks with the corresponding depth-normalized input count (Supplementary Fig. 34). Then, the estimated noise is subtracted from the depth-normalized IP counts in each genomic region, resulting in the background-noise normalized IP enrichment. Finally, the normalized enrichment signal was transformed with the inverse hyperbolic sin ($\boldsymbol{asinh}$):

$$\boldsymbol{asinh} = \boldsymbol{ln}\,(\boldsymbol{x} + \sqrt{\boldsymbol{x^2} + \boldsymbol{1}})$$

This transformation makes the data homoscedastic, that is, with approximately equal spreads despite pronounced variations in enrichment levels. This eases the handling and interpretation of the data and is fundamental in the regression models that will be implemented later on.

RNA-seq alignment and gene expression quantification

RNA-seq 101bp PE reads were aligned to the corresponding reference genome along with the genome of the Epstein-Barr virus using hisat2 (version 2.0.4)[8] with default parameters. Low-quality and multiple-mapping reads were removed using Samtools (version 2.1)[5] with options '-q30 -F 1804 -f 2'. Potential PCR duplicates were removed using PICARD v1.91 (http://picard.sourceforge.net). Read counts in genes were computed using htseq-count[9]. We used the R package DESeq2 (version 1.14.1)[10] to evaluate the coherence between technical replicates, exploring sample-to-sample distances and PCA results. Technical replicates were highly correlated and hence collapsed and used thereafter (Supplementary Fig. 35 and Supplementary Table 6). Gene quantification on merged replicates was performed using STRINGTIE (version 1.3.3)[8] based on species-specific Ensembl version 91[11] gene annotations. Estimated gene expression levels in TPM (transcripts per million transcripts) were obtained from the STRINGTIE/1.3.3.

Establishment of orthologous relationships

To find 1-to-1 orthologous genes, we mined the Ensembl[11] database was using biomaRt[12]. We retrieved the following features for the non-human species studied: Ensembl genes ID, gene coordinates, homology type and orthology confidence. Only autosomal one-to-one orthologous genes with the highest orthology confidence value (1) were kept for defining the set of 1-to-1 orthologous genes across the five species (11,249 genes). Of these, 9,936 were annotated as protein-coding in all species and 7,850 were expressed (TPM ≥ 0.5) in at least one species.

To find orthologous regulatory regions, we followed the approach described in Supplementary Fig. 36. First, we mapped non-human regulatory elements to the human reference genome and considered orthologous regulatory elements those with a minimum overlap of 50% and for which the overlapping regions had similar size (50% of each other) (Supplementary Fig. 36a). In the case of several overlapping regions, we prioritized the regions with the largest overlap. For every orthologous region for which we could not recover an orthologous regulatory element in at least one species, we defined the primate orthologous regulatory region coordinates as a genomic region of size equal to the average size of the species orthologous elements and located in the middle of the collapsed orthologous elements coordinates. We used these primate orthologous regions to recover overlooked orthologous relationships, this is, regulatory elements that either overlapped or were close (200 bp) to the primate orthologous region (Supplementary Fig. 36b). If no regulatory element was recovered, the coordinates of the primate orthologous region were mapped to the corresponding species assembly (Supplementary Fig. 36c). For species-specific regulatory elements for which we found no orthologous regulatory elements in any of the other species, we mapped their coordinates to the other species reference genome assemblies and considered those regions as the corresponding orthologous region (Supplementary Fig. 36d). All inter-species projections were performed using the liftOver tool from the UCSCTOOLS/331 suite[13]. We used pairwise best reciprocal chains. For every inter-species projection, coordinates were mapped twice, going forward and backward, and only regions that could be properly mapped in both directions were kept. Overlaps were performed using the intersectBed tool from the BEDTools suite[14].

Note here that our dataset of orthologous regulatory regions is restricted to genomic regions that can be mapped across species (regions for which an orthologous region at the sequence level can be found). This is a restrictive protocol that ensures the comparability and balance between different species, which have very different levels of annotation and genome assembly qualities. However, this comparability comes at the price of not being able to study the epigenomic changes associated with genomic gains and losses. To assign a regulatory state to those orthologous regions not associated with a regulatory element, we used the underlying enrichments in histone modifications and open chromatin (see Assignment of a regulatory state to regulatory elements). For those analyses comparing the regulatory state at orthologous regulatory regions associated with genes, we assigned a consensus regulatory

component type to each orthologous regulatory region considering the different quality of the assemblies and gene annotations. Specifically, we assigned to the group of orthologues the type of regulatory component assigned in more species. In the case of a draw, we established the following hierarchy: human > chimpanzee > macaque > gorilla > orangutan.

Normalization of gene expression and enrichment signals across species

First, we used batch correction to remove the technical variation derived from the integration of previously published data for the cell line GM12878. The batch effect was corrected using the ComBat function implemented in the R package sva (version 3.22.0)[15]. Then, we developed a method to normalize gene expression across samples. This method is based on the identification of a set of internal reference controls (IRCs) (genes) whose expression is constant across samples and from which a normalization factor can be derived (Supplementary Fig. 37). We defined internal reference controls as genes that were not outliers in any pairwise sample comparison, where outliers were defined as any gene with pairwise sample differences larger than the pairwise sample-specific median difference plus two median absolute deviations. We first standardized (robust standardization) each sample signal in IRCs. Then, we defined the representative IRCs values as the means of the standardized values of each IRC in the different samples. These representative IRCs are used to define a common scale for all the samples. We modeled the linear relationship between IRCs of each sample and the representative IRC values using Deming regressions. Sample-specific normalization factors are the result of dividing each sample-specific slope by the mean slope of all samples. To normalize the signal of all genes, we first scaled each sample signal in the robustly standardized distribution of IRCs using the corresponding sample median and median absolute deviation computed using these standardizations. Then, we proceeded to do the normalization with the corresponding sample normalization factor. Finally, to retrieve normalized signals, destandardization was performed using the mean and standard deviation of the samples' IRC median and median absolute deviations.

To assess the performance of the developed calibration method, we evaluated the effect of both the batch correction and expression signal normalization for every sample-pairwise comparison. To do so, we measured the angle to the identity line (x=y). If the normalization procedure removed noisy variance effectively, differences between samples were expected to be reduced. This would reflect in regression lines closer to the identity line (Supplementary Fig. 38). Our method effectively removed technical noise in the expression signal, the angles were successfully shrunk both at IRCs and pairwise orthologous expressed protein-coding genes excluding IRCs (Supplementary Fig. 39).

We further compared our normalization method with the widely used quantile normalization[16] and found that our method outperformed the latter, particularly at normalizing the tails of the distributions

(Supplementary Figs. 40 and 41). Normalization of the tails is a known problem associated with quantile normalization, which cannot properly handle the greater inter-samples differences at extreme values. Our parametric approach overcomes this limitation and correctly calibrates signal values throughout the whole distribution.

We applied the same method to normalize the enrichment signals of the histone modifications at regulatory elements associated with orthologous protein-coding genes.

Analysis of whole-genome sequencing data (WGS)

WGS mapping

151 bp PE reads were mapped to the corresponding species reference genome assemblies: human (hg38), chimpanzee (panTro5), gorilla (gorGor4), orangutan (ponAbe2) and macaque (rheMac8) along with the reference genome of the Epstein-Barr virus. Read alignments were carried out using BWA-MEM (version 0.7.8-r455)[17] with default parameters. Low-quality and multiple-mapping reads were removed using Samtools v1.6[5] with options '-q30 -F 1804 -f 2' and potential PCR duplicates were removed using PICARD v1.91 (http://picard.sourceforge.net).

Genotyping

WGS was used for genotyping every cell line and hard-filtering criteria were applied to identify SNPs, Indels and STRs. Variant discovery was performed using GATK version v3.7[18]. 'HaplotypeCaller' was run for each sample independently with default parameters using the '--emitRefConfidence GVCF' mode. Subsequently, both biological replicates were jointly genotyped using 'GenotypeGVCFs'. Hard filtering was conducted following GATK's recommendations. For SNPs, positions were removed if: 'QD < 2', 'MQ < 40', 'FS > 60', 'SOR > 3'. For Indels, positions were removed if: 'QD < 2', 'FS > 200', 'SOR > 10'. We further filtered variants based on their sample-specific callability, determined with the tool 'CallableLoci' with the following parameters: --minDepth 4, --maxDepth 100. Finally, mappable variants were kept based on 151-kmers species-specific mappability tracks.

We performed principal component analysis (PCA) with EIGENSOFT (version 7.2.1)[19] using single nucleotide variants on chromosome 21 to evaluate how our samples related to the great ape individuals genotyped in the GAGP2[20]. Variants were mapped to hg38 coordinates and chromosome 21 variants were selected for the analysis. For the mitochondrial PCA, sequences from several studies were compiled to create a mitochondrial reference panel (Supplementary Data 20). Each sequence was mapped to hg38 mitochondrial sequence with bwa-mem[9] and variants were called with VarScan2[21]. The obtained clusterings confirmed the species of the samples included in this study (Supplementary

Fig. 42) and further allowed us to infer the corresponding subspecies of individual non-human great ape samples (Supplementary Data 21). Macaque samples were of known Burmese origin. In addition, the sample-specific X chromosome coverage was used to confirm the sex of every sample included in the study (Supplementary Data 21). The number of reported SNPs, Indels and STRs fall within the expected range considering the species-specific genomic diversity and the inferred subspecies (Supplementary Data 21). Short tandem repeats (STRs) were called using HipSTR[22] with default stutter models and setting to 15 the minimum number of reads required to genotype a locus. STR filtering was performed using the recommended parameters: --min-call-qual 0.9 --max-call-flank-indel 0.15 --max-call-stutter 0.15 --min-call-allele-bias -2 --min-call-strand-bias -2.

Analysis of whole-genome bisulfite sequencing data (WGBS)

Bisulfite converted sequencing data was used to estimate CpG methylation values genome-wide. Individual CpG methylation levels are inferred as the fraction of aligned methylated reads. In this regard, it is important to note the impact of nucleotide variants, genomic positions where the reference genome and the sequence of the sample under study differ. SNPs can introduce biases due to incorrect estimation of the methylation state: heterozygous loci might be assigned intermediate methylation values, whereas homozygous sites would result in an unmethylated call. Matching whole-genome sequence data allows the avoidance of this bias.

151 bp PE reads were mapped to the *in silico* bisulfite-converted species reference genome assemblies along with the *in silico* bisulfite-converted genome of the Epstein-Barr virus using Bismark (version 0.16.1)[23]. Low-quality and multiple-mapping reads were removed using Samtools (version v1.2)[5] with options '-q30 -F 1804 -f 2'. Potential PCR duplicates were also removed using Bismark's deduplicate_bismark program. Inspection of M-bias plots[24] revealed a methylation bias towards the end of the reads, so measurement at the last 15 positions was excluded from further analysis.

Custom Bash scripts were used to filter mappable CpG sites based on 151-kmers species-specific mappability tracks. Individual CpG methylation levels were summarized for each sample computing the ratio of unmodified Cs (methylated) to bisulfite converted Ts (unmethylated) using the R package bsseq (version 1.8.2)[25]. Only autosomal CpGs with a minimum of 4x coverage were considered for downstream analysis.

On average, a CpG coverage of ~16X was achieved after filtering CpGs located in non-mappable species-specific regions, removing sample-specific identified polymorphic CpGs and setting a minimum threshold of 4 sequencing reads. Methylation values were then inferred in approximately 17.4 million CpG sites per sample. Overall, the five species exhibited similar levels of CpG methylation,

with an average value of ~61%. These values are expected and comparable to previous estimates in LCLs[26], which are, in general, lower than those observed in non-transformed tissues, e.g., ~72% global methylation in great-ape blood[27]. Nonetheless, orangutan sample O1 stood out, due to its unusually low global methylation (average sample-specific methylation value of 48.6%). As a result, Pearson correlation values between orangutan replicates were much lower (64%) than among human (81%), chimpanzee (77%), gorilla (77%) and macaque (81%) samples.

The R package MethylseekR (version 1.12.0)[28] was used to identify unmethylated regions (UMRs) and low methylated regions (LMRs). Methylation levels and FDR parameters were inferred from the data, as suggested by the MethylseekR workflow.

UMRs tend to be CpG-rich regions and are commonly regarded as proximal regulatory elements; LMRs, CpG-poor, have been associated with the binding of transcription factors, which cause the local reduction of otherwise high methylation levels, and are largely considered as distal regulatory elements, highly dynamic and tissue-specific[29]. Overall, similar numbers of UMRs and LMRs were found across samples and species (Supplementary Fig. 43). Notably, sample O1 showed a discordant pattern, with particularly elevated numbers of both UMRs and LMRs.

The relationship between UMRs and LMRs and chromatin states was explored, and the expected trends were observed. On the one hand, UMRs showed an enrichment pattern highly akin to that of CGI. On the other hand, LMRs overlap with active intergenic enhancer states (chromatin states E5, E6 and E7, Supplementary Figs. 2 and 33 and Supplementary Data 22).

Supplementary Figures

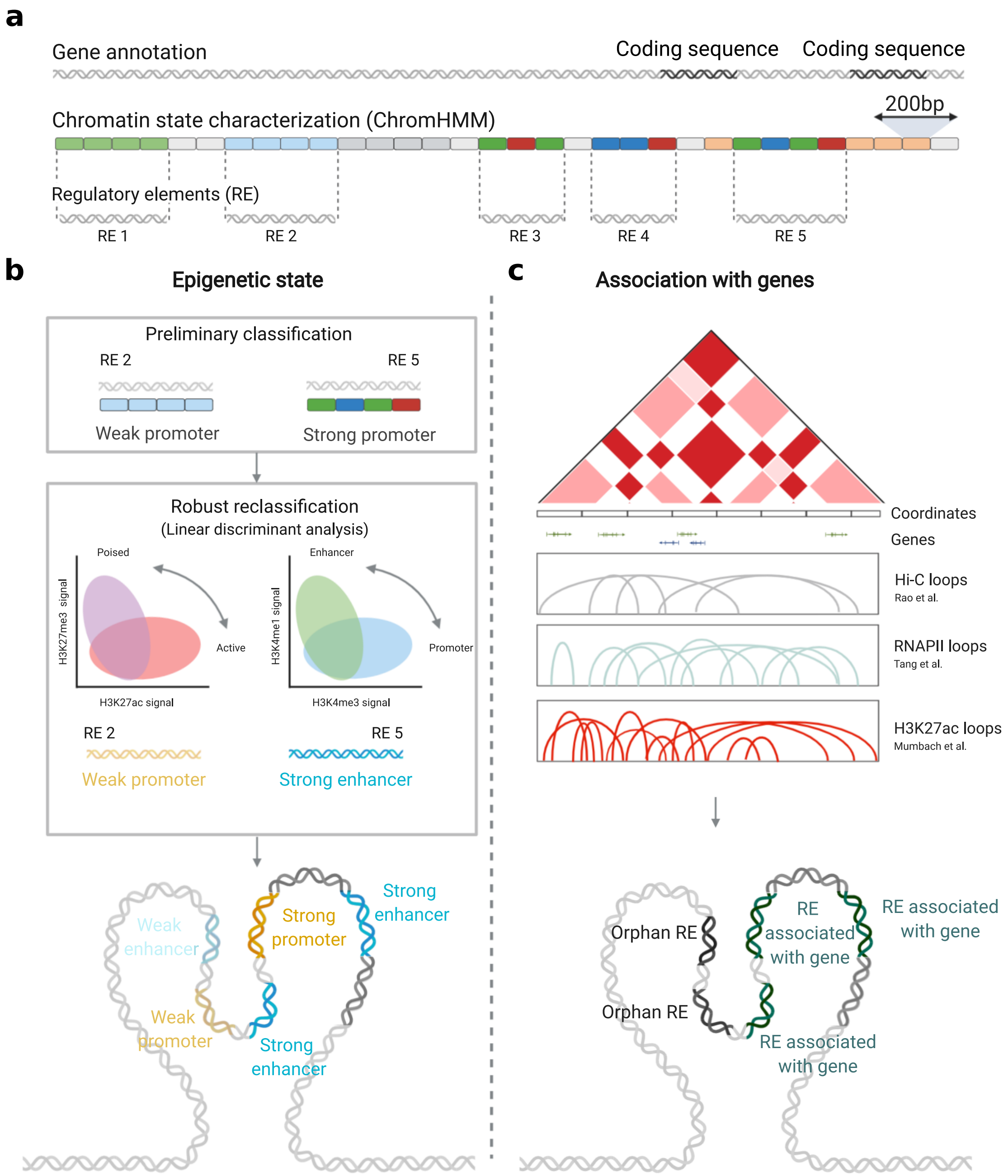

Supplementary Figure 1. Schematic illustration of the approach followed to annotate and classify regulatory elements. a, DNA strand represents a gene annotation track, wherein dark grey regions correspond to coding annotated regions. The second row represents the binarized output from ChromHMM, wherein each box corresponds to a 200 bp bin. Light grey indicates bins without evidence of promoter or enhancer states, whereas the different colors represent the different learned chromatin states. Shorter DNA strands represent the genomic coordinates defined for regulatory elements that result from merging adjacent 200 bp bins with epigenetic signals associated with promoter or enhancer states. We defined species regulatory elements from the union of the regulatory elements detected in each biological replicate. **b,** We

established a hierarchy between chromatin states based on the combination of chromatin marks found within each regulatory region and classified regulatory elements into epigenetic promoter (P) and enhancer (E) states with three different activity levels: strong (s), weak (w) or poised (p). Then, we applied a linear discriminant analysis (LDA) using normalized histone and open chromatin enrichments to refine this epigenetic classification. **c,** We linked regulatory elements to genes based on gene proximity and using previously published 3D chromatin maps in GM12878 cells, we recovered physical interactions between regulatory elements. We additionally assigned regulatory elements associated with genes to a type of regulatory component. We classified regulatory elements into genic promoters (gP), genic enhancers (gE), proximal enhancers (prE), promoter-interacting enhancers (PiE) and enhancer-interacting enhancers (EiE) (Fig. 4a) (Methods and Supplementary Methods, Supplementary Fig. 2).

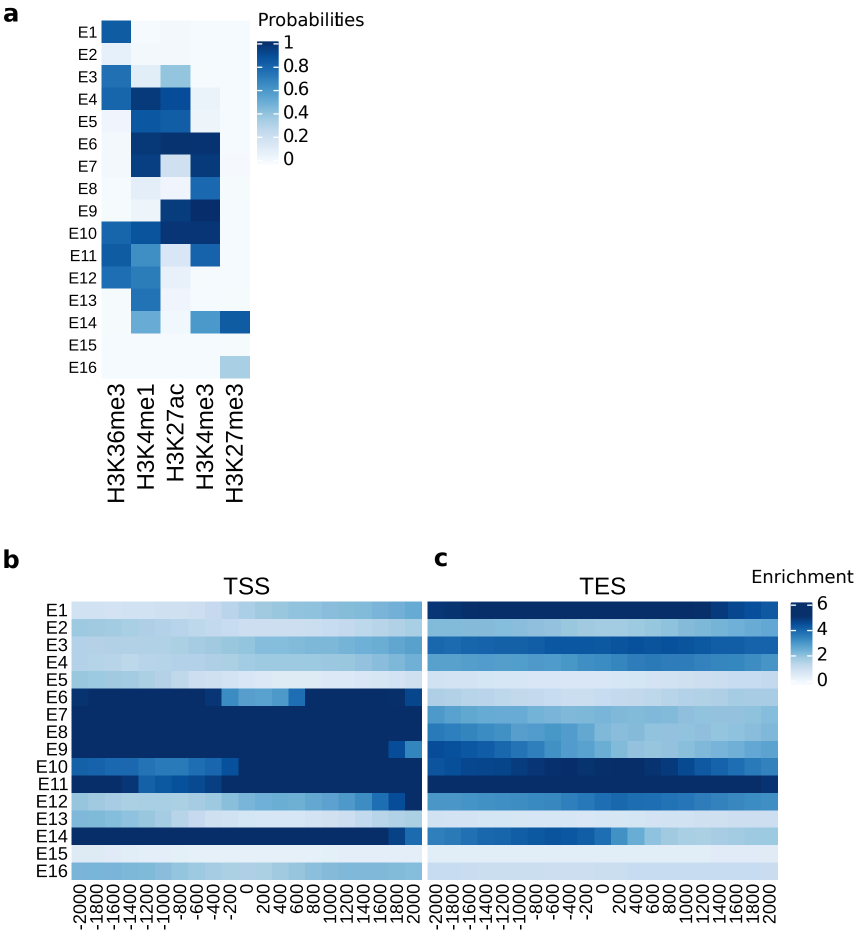

Supplementary Figure 2. Chromatin states are enriched in biologically meaningful genomic regulatory regions. Each row corresponds to one of the 16 chromatin states learned using the combined information of five histone marks (H3K4me3, H3K4me1, H3K27ac, H3K36me3 and H3K27me3). **a,** Emission parameters from ChromHMM. Cell intensity determines the probability of observing a specific mark in a given state. **b,** and **c,** Overlap and neighborhood enrichment analyses in functionally defined regions. Cell values represent the average fold enrichment across samples over different functional annotations and distances from **(b)** transcription start sites (TSS) and **(c)** transcription end sites (TES). The 16 chromatin states recover major regulatory regions, including active promoters (E8, E9) and enhancers (E6) states, flanking upstream and downstream promoters (E7, E11) and enhancers (E5, E10), bivalent states (E14), elongation states (E1 and E2), heterochromatin (E16) and low signal (E15). Chromatin states do not reach the proper resolution to distinguish between poised promoters and enhancers, although both types of regulatory elements are present in the data (E14).

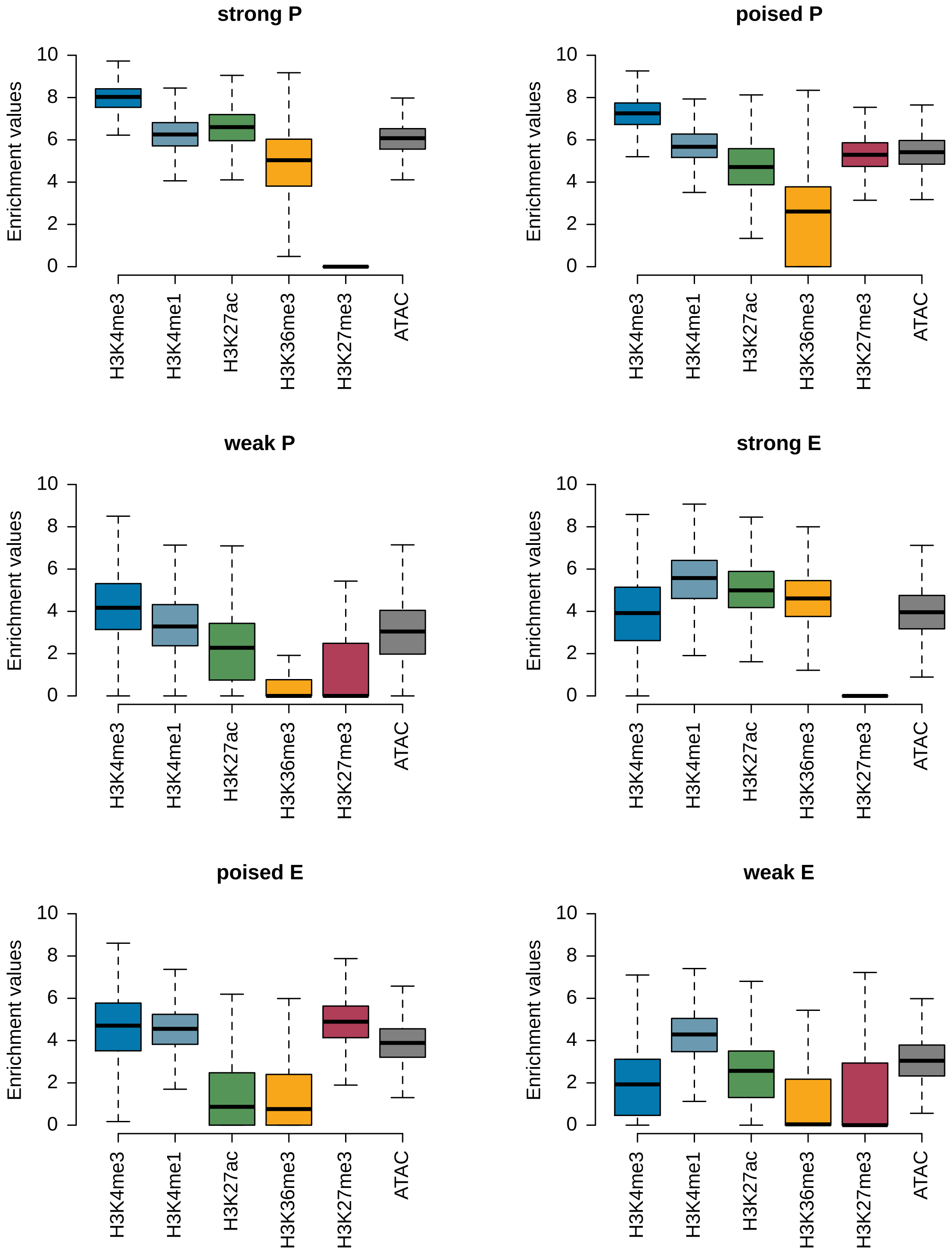

Supplementary Figure 3. Distribution of normalized enrichment values associated with histone marks and open chromatin signals in each regulatory state across samples. Box plots show medians and the first and third quartiles (the 25th and 75th percentiles), respectively. The upper and lower whiskers extend the largest and smallest value no further than 1.5 × IQR (n = 89,547 for strong P; n = 17,503 for poised P; n = 37,707 for weak P; n = 323,821 for strong E; n = 52,036 for poised E; n = 452,570 for weak E).

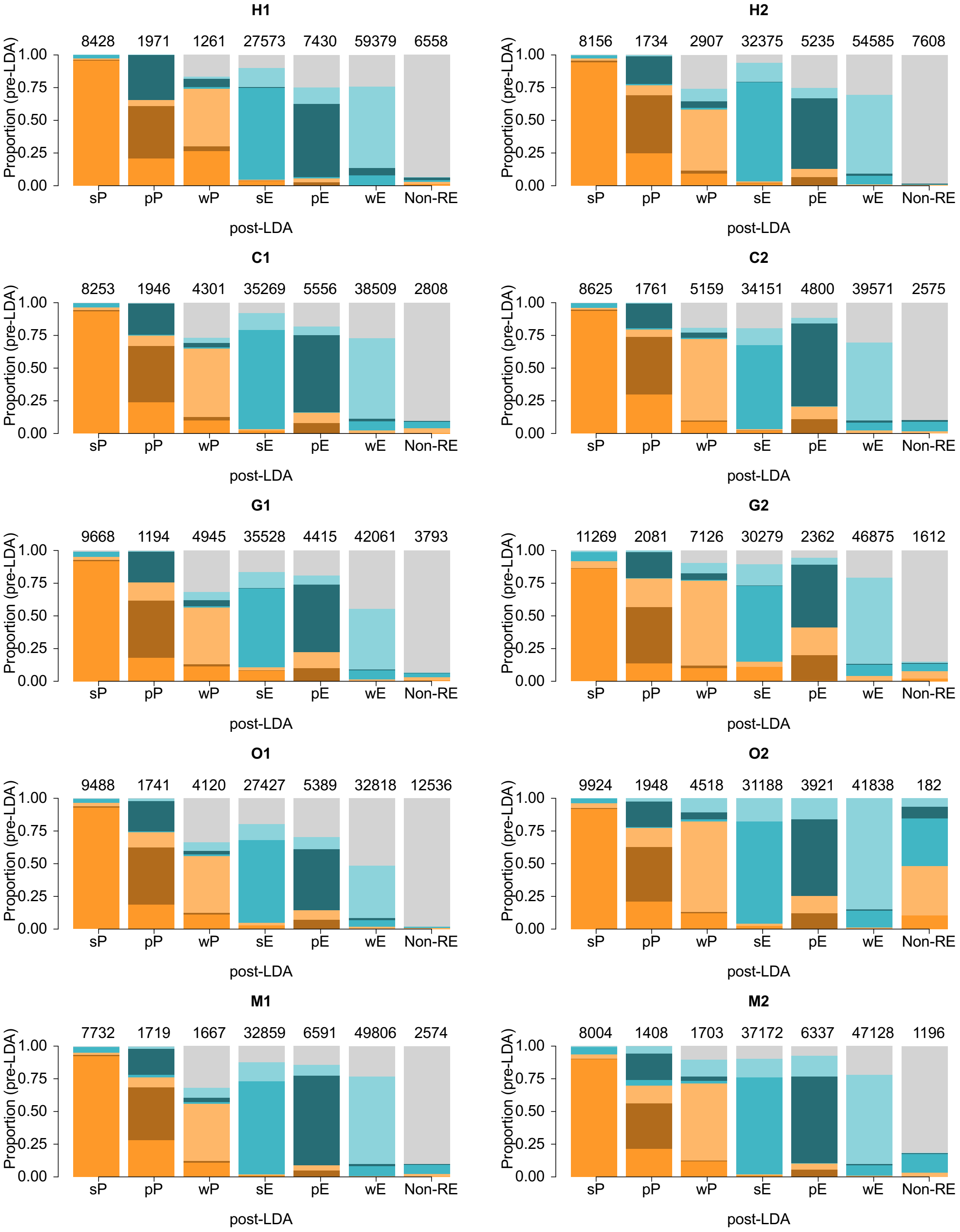

Supplementary Figure 4. Linear discriminant analysis (LDA) of the regulatory elements. Each bar plot shows, for each species and regulatory state, the proportion of post-LDA assignments compared to its pre-LDA assignment. Numbers on top of the bars indicate the number of regulatory elements in each regulatory state. Regulatory states are color-coded as in Fig. 2 and grey indicates non-regulatory elements.

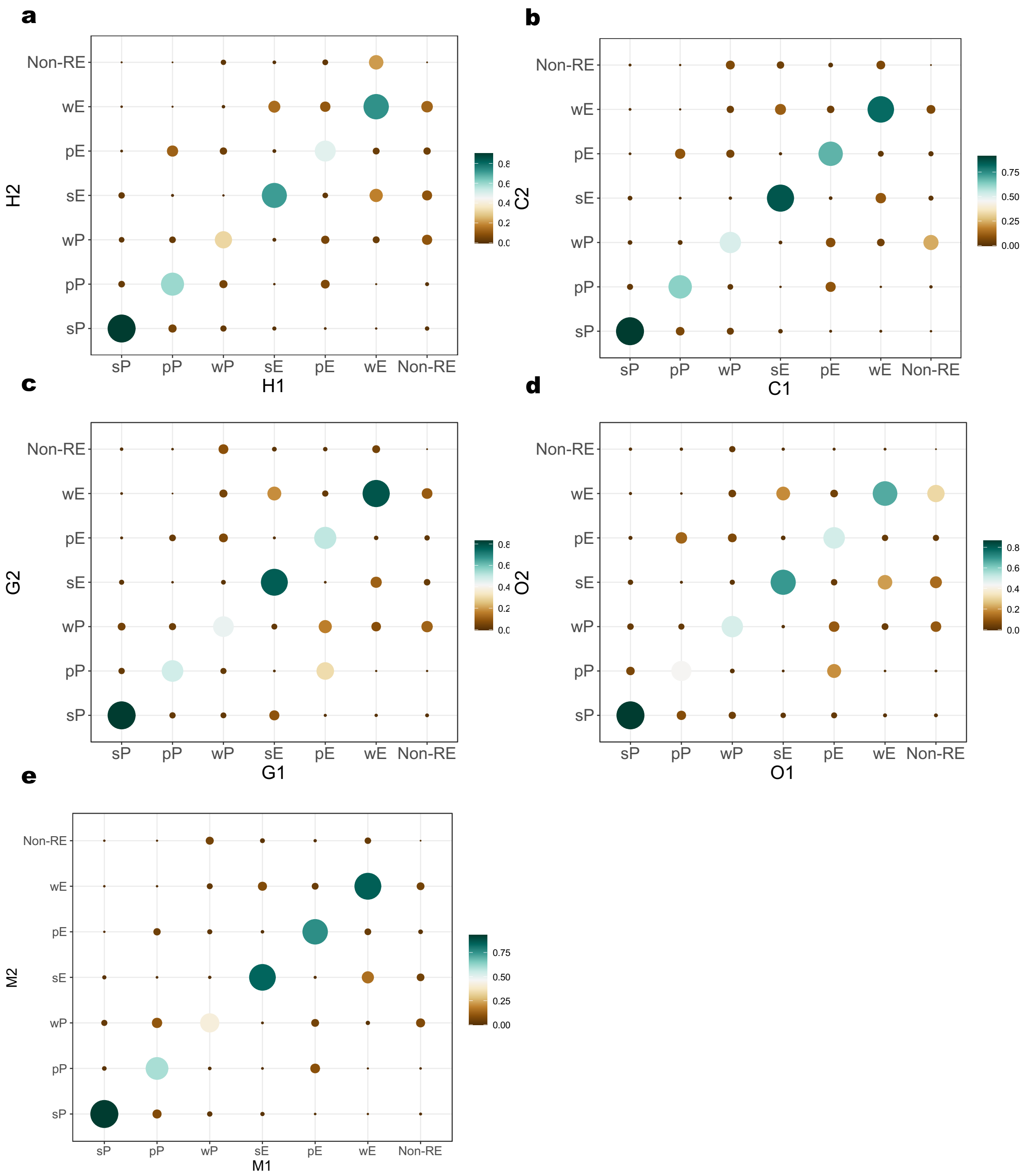

Supplementary Figure 5. Similarity in the regulatory states assigned to regulatory elements between biological replicates. To evaluate the similarity in the regulatory states between biological replicates, we calculated the Sørensen-Dice similarity coefficient (DSC) as DSC=2·x∩y/x+y wherein x and y are the numbers of regulatory elements with a given regulatory state in each of the replicates. **a-e,** Similarity between human, chimpanzee, gorilla orangutan and macaque replicates, respectively. The DSC scores associated with regulatory elements with the same regulatory state in both biological replicates (diagonal) have higher values after the linear discriminant analysis (one-tailed Wilcoxon signed rank-test; $P < 0.05$ in all species).

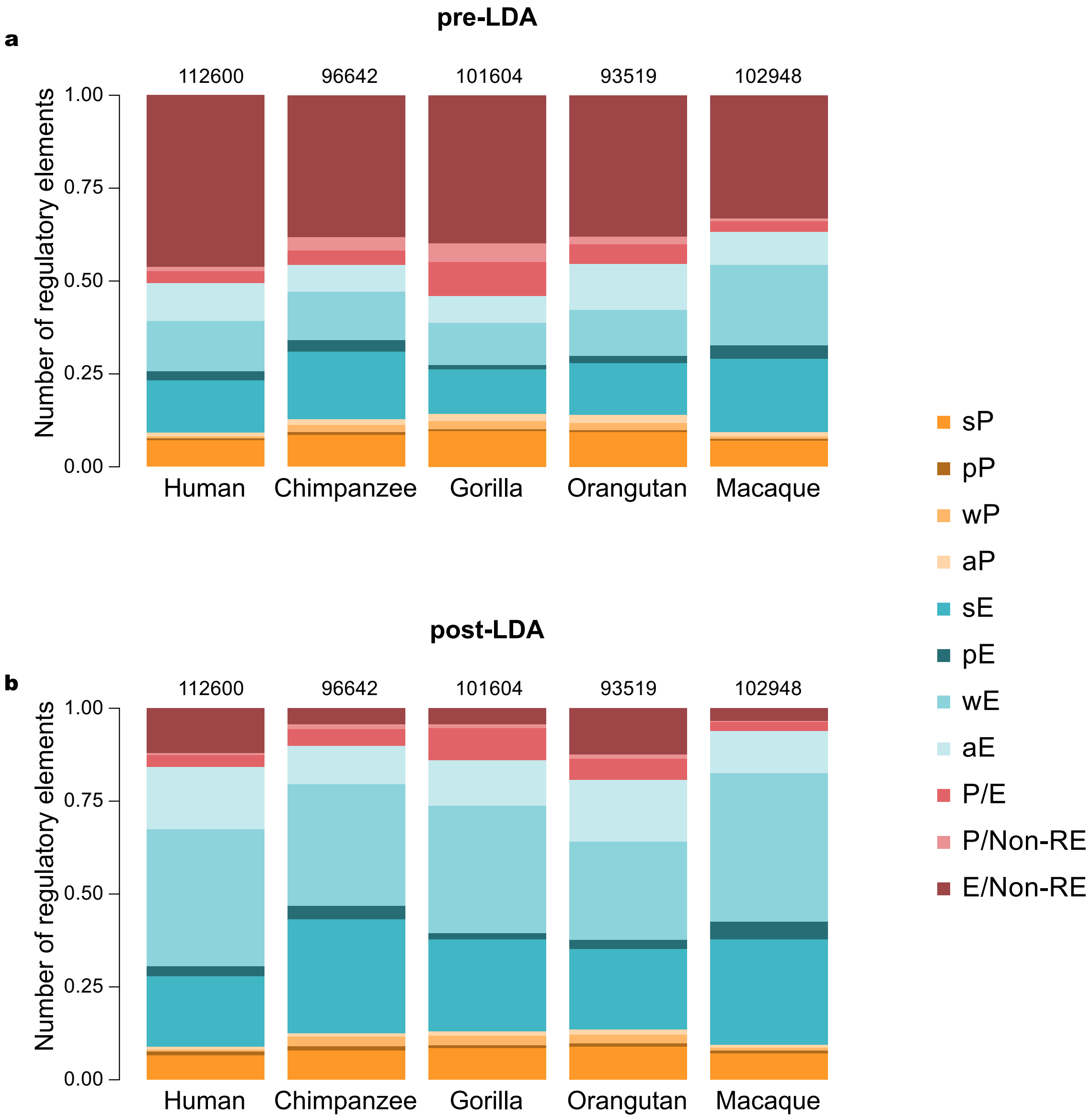

Supplementary Figure 6. Comparison of the regulatory state of the regulatory elements before and after the linear discriminant analysis (LDA). Each bar plot shows the proportion of regulatory elements with a given regulatory state. Numbers on top of the bars indicate the total number of regulatory elements annotated per species. Color-coded the different regulatory states **a,** pre-LDA composition; **b,** post-LDA composition. aP and aE correspond to regulatory elements with consensus states (both promoters or enhancers, respectively) but with different activities between replicates (e.g., sE in one replicate and pE in the other replicate). P/E correspond to regulatory elements with a promoter state in one replicate and an enhancer state in the other replicate. P/Non-RE and E/Non-RE correspond to regulatory elements with a promoter or enhancer state in one replicate and no evidence of regulatory activity in the other replicate.

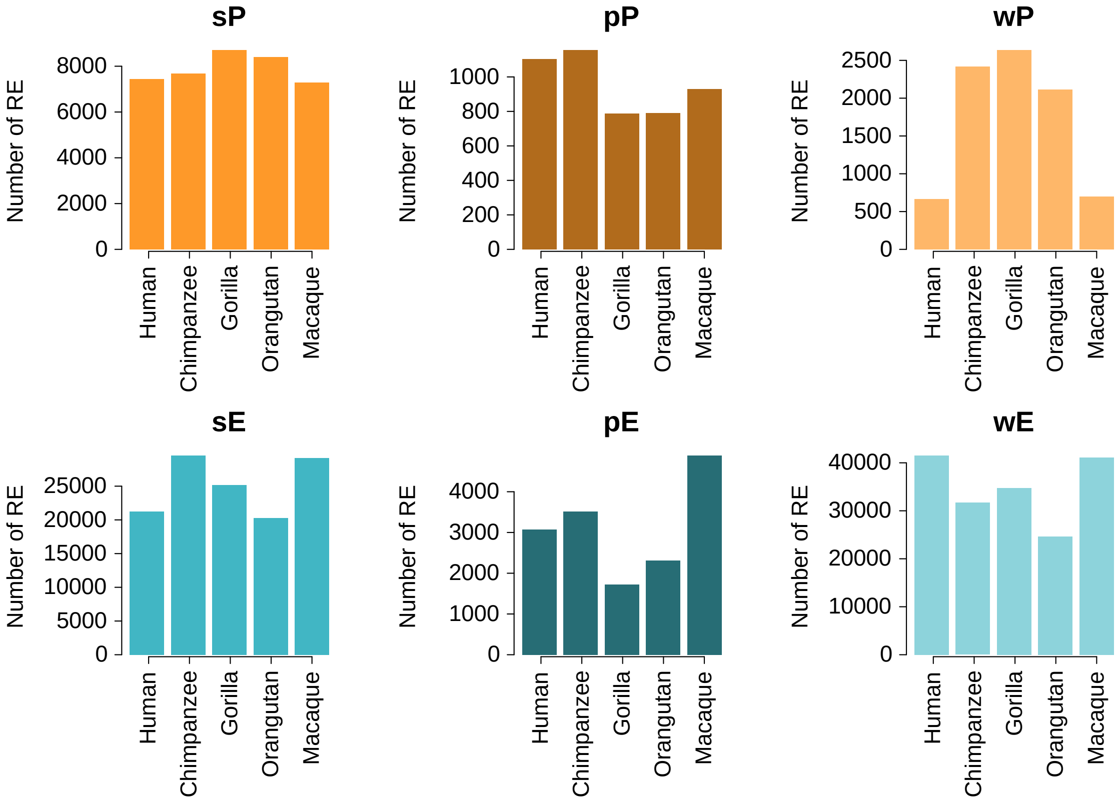

Supplementary Figure 7. Number of regulatory elements with the same regulatory state between replicates in every species. Regulatory states are color-coded as in Fig. 2. (P: promoter; E: enhancer; s:strong; p:poised; w:weak)

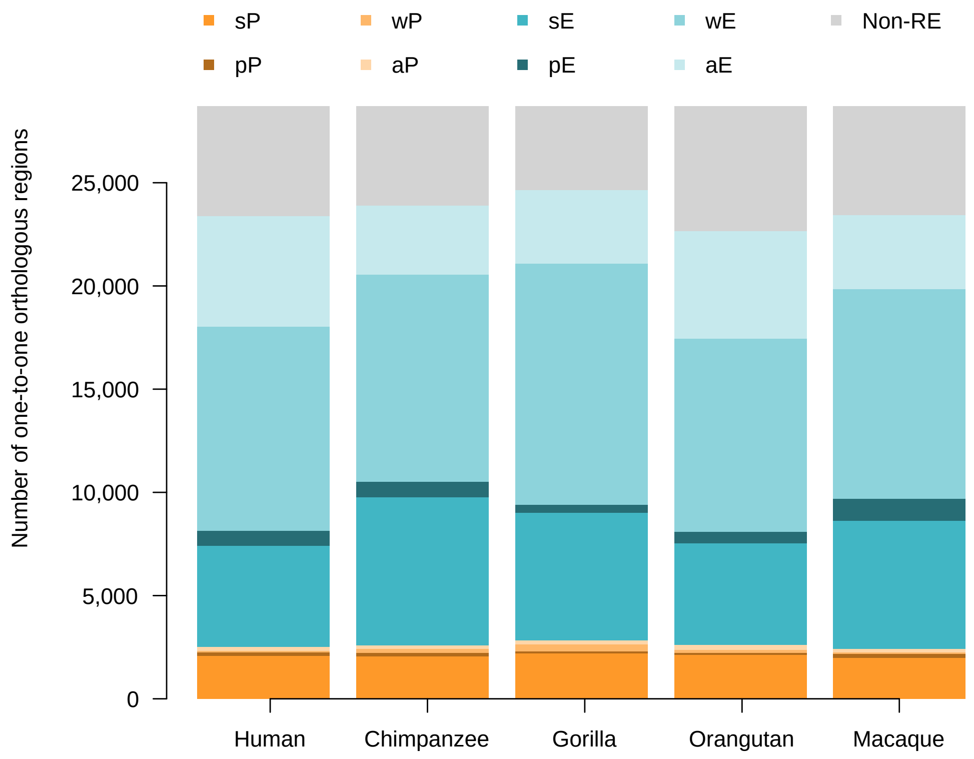

Supplementary Figure 8. Regulatory states at orthologous regulatory regions. Each bar plot shows, for each species, the number of orthologous regulatory regions with the corresponding color-coded regulatory state.

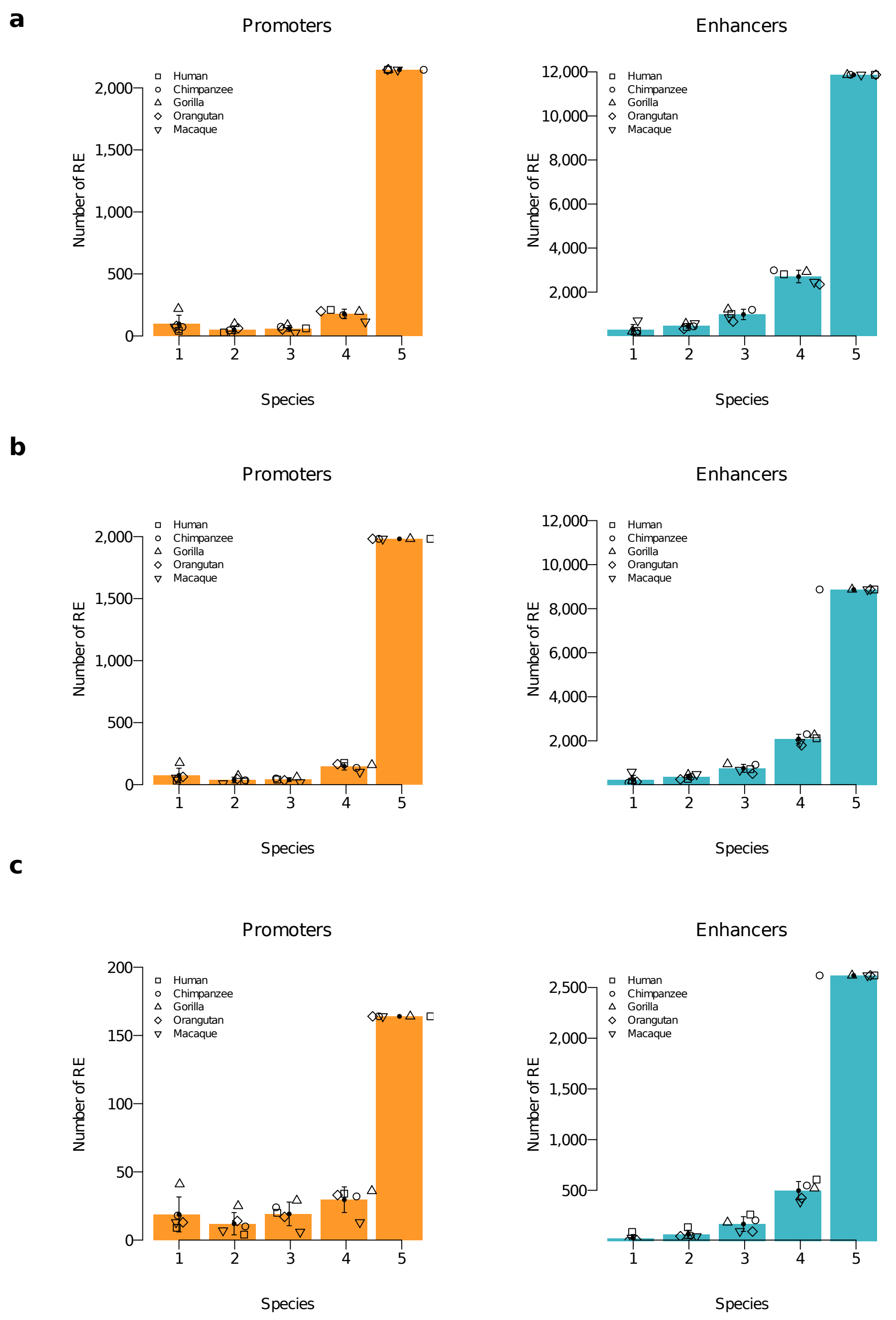

Supplementary Figure 9. Promoter states at orthologous regulatory regions are more evolutionarily conserved than enhancer states. Bar plots show the average number of orthologous regulatory regions with a promoter or enhancer state in 1, 2, 3, 4 or 5 species. **a,** Orthologous regulatory regions associated with genes (two-tailed Fisher's exact test; $P = 1.02 \times 10^{-21}$, $OR = 1.48$). **b,** Orthologous regulatory regions associated with human protein-coding genes (two-tailed Fisher's exact test; $P = 5.27 \times 10^{-42}$, $OR = 1.87$). **c,** Orthologous regulatory regions associated with human non-coding genes (two-tailed Fisher's exact test; $P = 7.38 \times 10^{-16}$, $OR = 0.39$). Points indicate average values and the error bars represent the s.d. (n = 5 species).

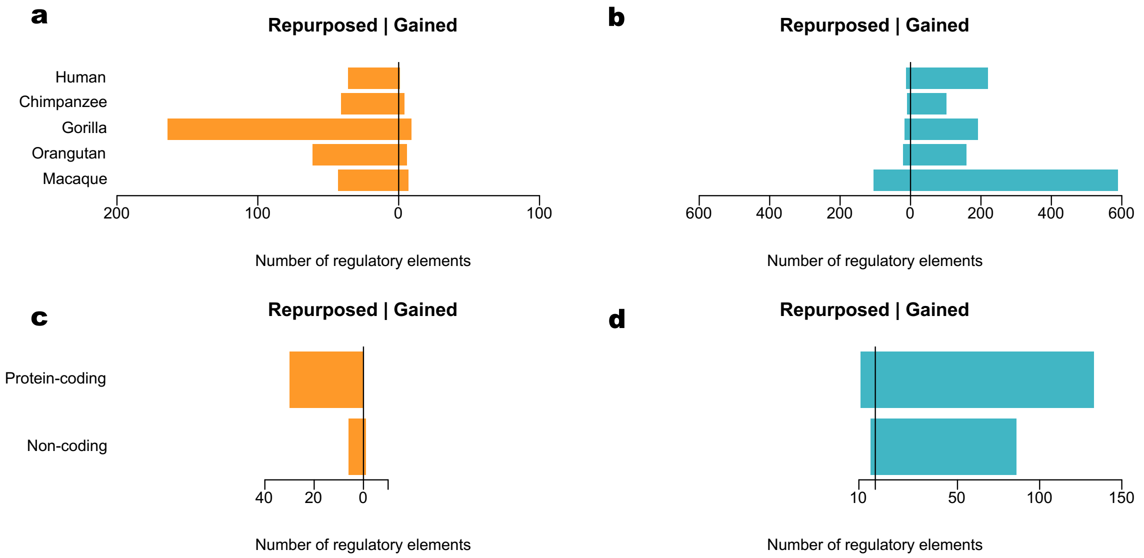

Supplementary Figure 10. Promoters originate via repurposing events, whereas enhancers emerge from orthologous regions without prior regulatory activity. Bar plots show the number of orthologous regulatory regions with promoter or enhancer states gained through repurposing events (left-side bar plots) or acquired *de novo* in orthologous regions (i.e., with no regulatory activity in the other species, right-side barplots). Species-specific **a,** promoters and **b,** enhancers associated genes. Human-specific **c,** promoters and **d,** enhancers associated with human protein-coding and non-coding genes.

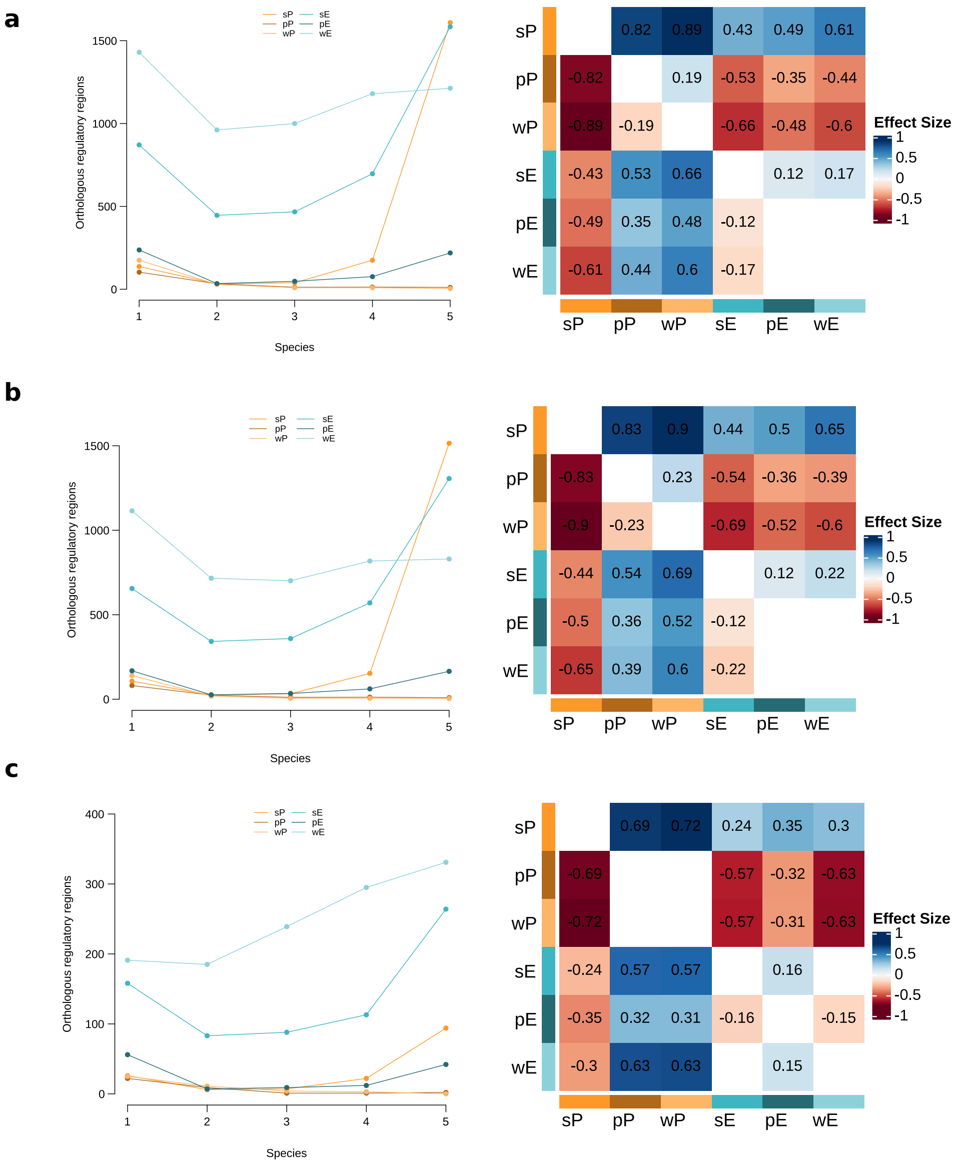

Supplementary Figure 11. Evolutionary dynamics of regulatory states. Left scatterplots show the patterns of evolutionary conservation for the different regulatory states. Right panels show the effect sizes of the pairwise comparison of the evolutionary conservation patterns between any two regulatory states (Dwass-Steel-Critchlow-Fligner test). Only the effect sizes of significant comparisons are shown ($P < 0.05$). The analyses are restricted to **a,** orthologous regulatory regions associated with genes (Kruskal-Wallis test; $P = 0$); **b,** orthologous regulatory regions associated with human protein-coding genes (Kruskal-Wallis test; $P = 0$) and **c,** orthologous regulatory regions associated with human non-coding genes (Kruskal-Wallis test; $P = 3 \times 10^{-26}$).

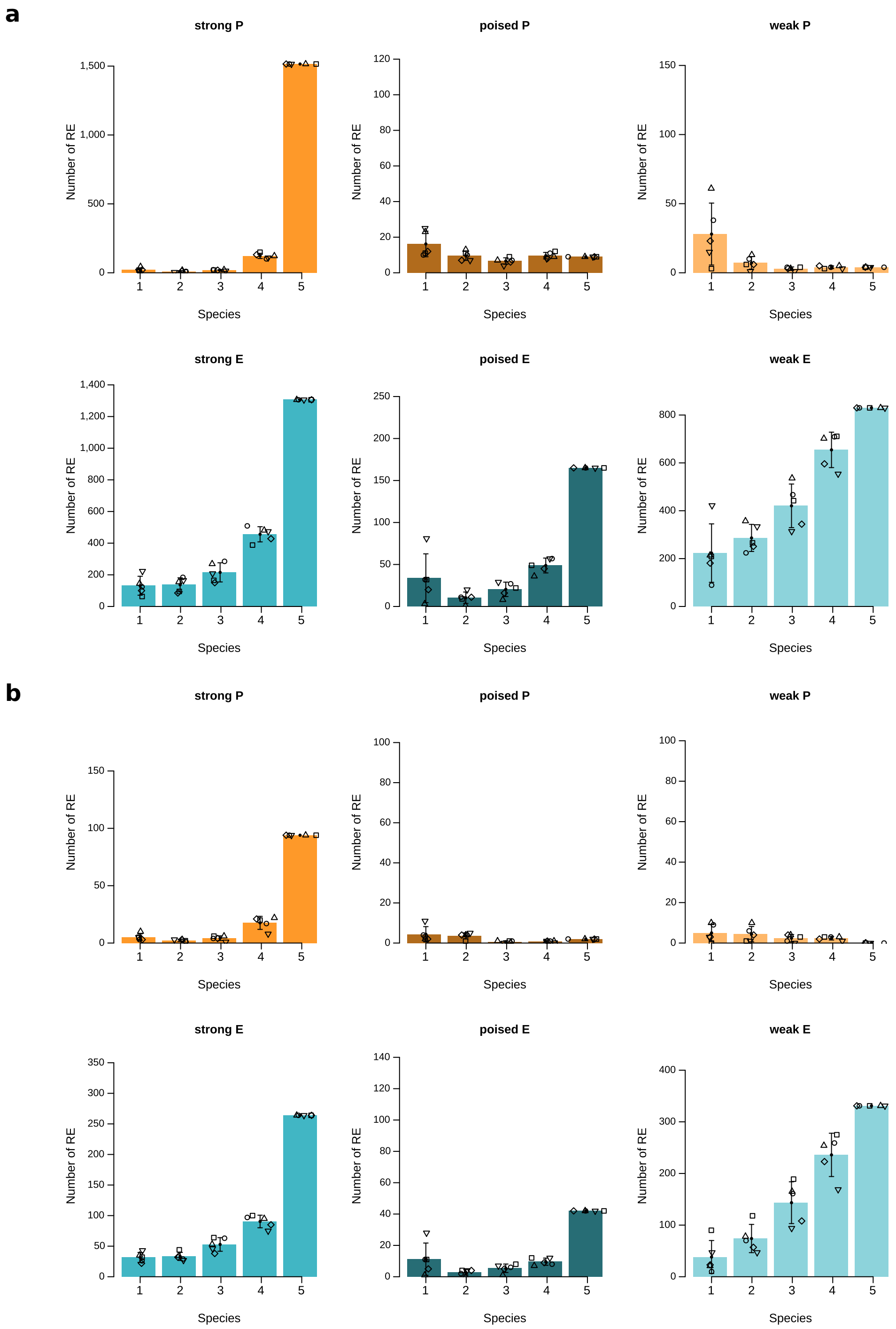

Supplementary Figure 12. Patterns of evolutionary conservation of each regulatory state. **a,** Orthologous regions associated with human protein-coding genes. **b,** Orthologous regions associated with human non-coding genes. Bar plots show the average number of orthologous regulatory regions across species with the corresponding color-coded epigenetic state conserved in 1, 2, 3, 4 or 5 species. Differently shaped dots show the number of regulatory regions with this conservation for each species. Points indicate average values and the error bars represent the s.d. (n = 5 species).

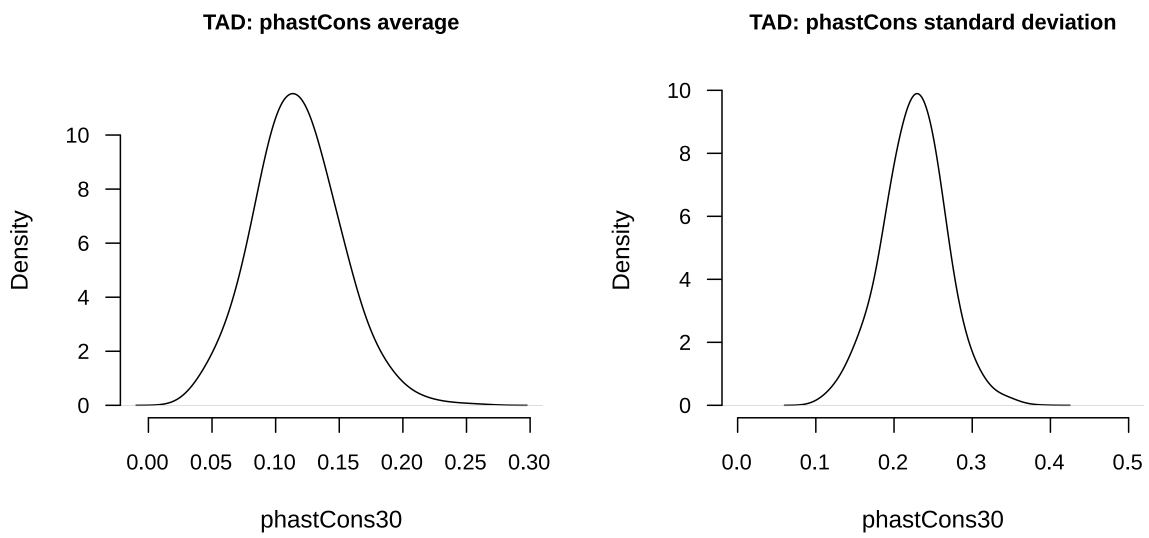

Supplementary Figure 13. Sequence conservation of TADs. Distribution of the average and standard deviation of the phastCons30 scores associated with TADs defined for the human cell line GM12878.

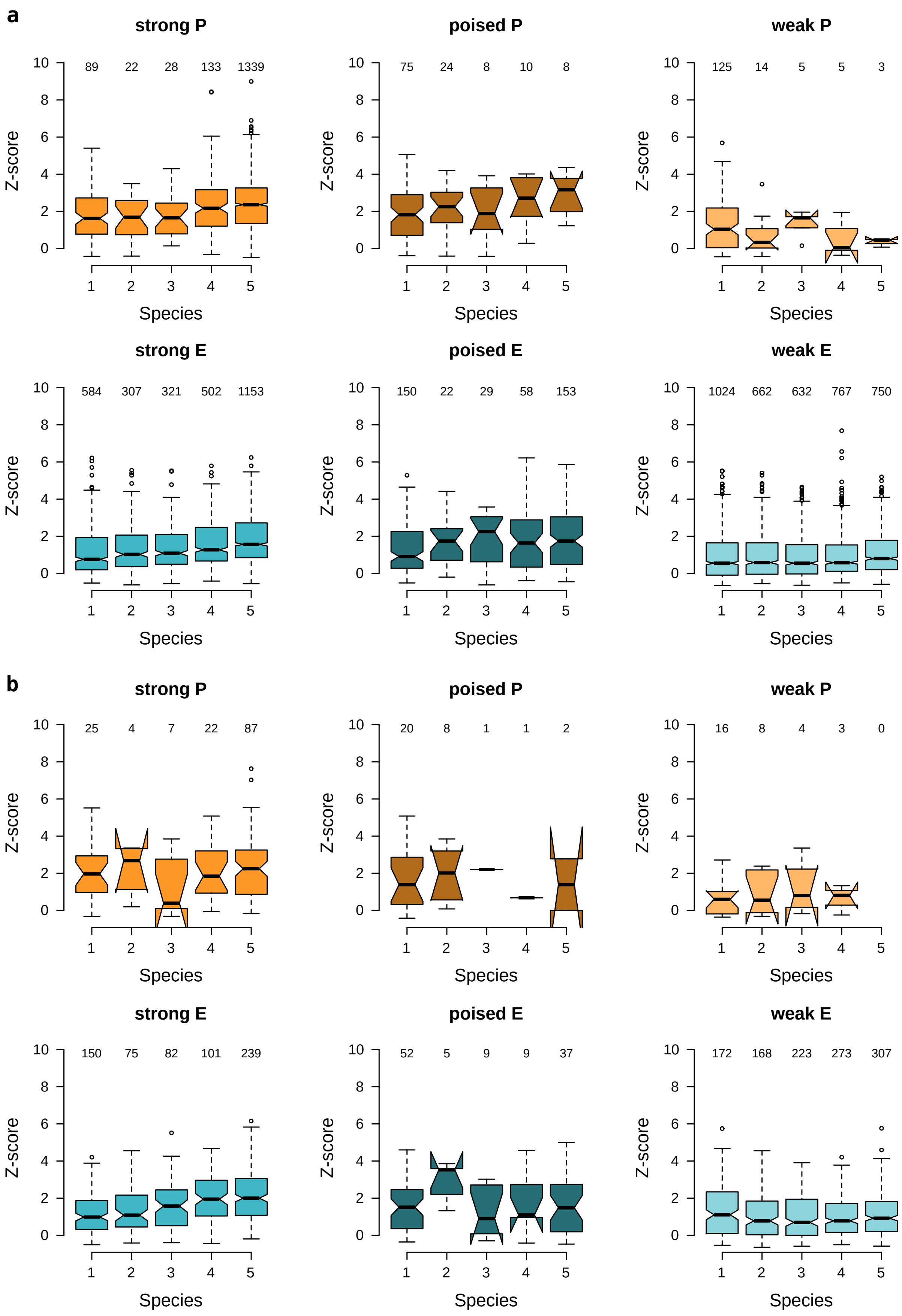
a
strong P
89 22 28 133 1339
poised P
75 24 8 10 8
weak P
125 14 5 5 3
strong E
584 307 321 502 1153
poised E
150 22 29 58 153
weak E
1024 662 632 767 750
b
strong P
25 4 7 22 87
poised P
20 8 1 1 2
weak P
16 8 4 3 0
strong E
150 75 82 101 239
poised E
52 5 9 9 37
weak E
172 168 223 273 307
Z-score
Species
1 2 3 4 5
0 2 4 6 8 10

Supplementary Figure 14. Distribution of sequence conservation scores of orthologous regulatory regions with different epigenetic states conserved in 1,2,3,4 or 5 of our primate species. Orthologous regulatory regions associated with **a**, human protein-coding genes or **b**, human non-coding genes. Box plots show medians and the first and third quartiles (the 25th and 75th percentiles), respectively. The upper and lower whiskers extend the largest and smallest value no further than 1.5 × IQR.

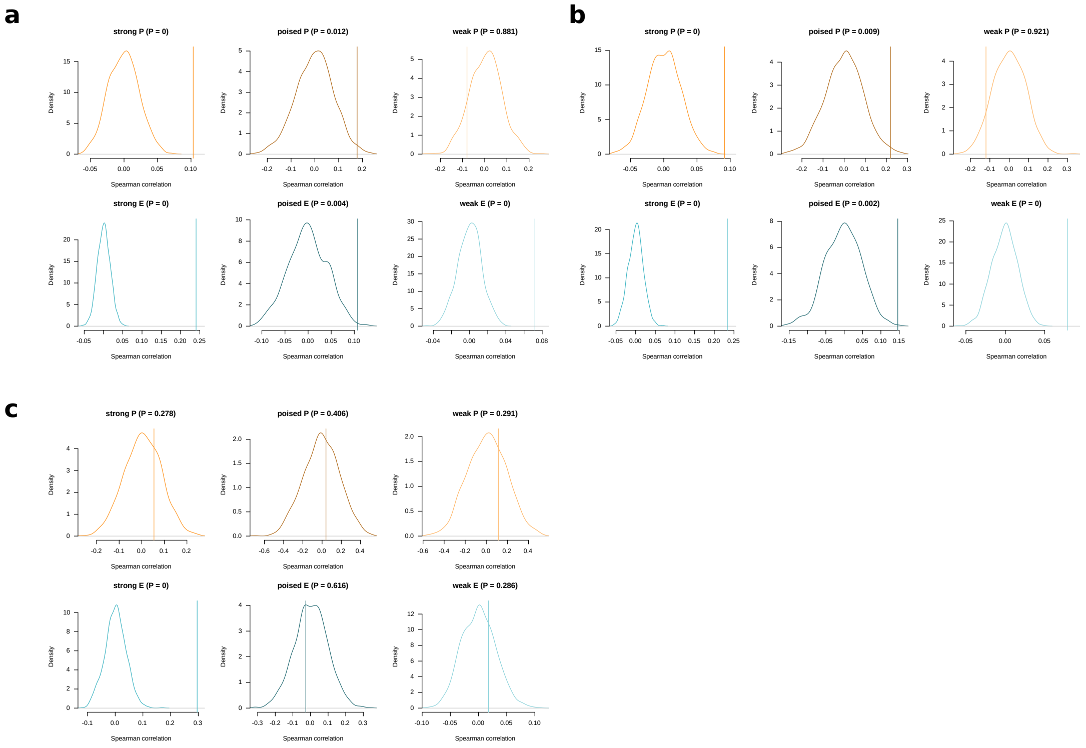

Supplementary Figure 15. The evolutionary conservation of the regulatory state in orthologous regulatory regions is statistically significant and positively correlated with the evolutionary conservation of the underlying sequence. Orthologous regulatory regions associated with **a,** genes; **b,** human protein-coding genes and **c,** human non-coding genes. The correlation is only significant for strong enhancer states in **c**. Density plots show the distribution of the Spearman's rank correlation correlation ρ values obtained in 1,000 randomizations. Vertical lines indicate the observed Spearman's rank correlation ρ value of our data.

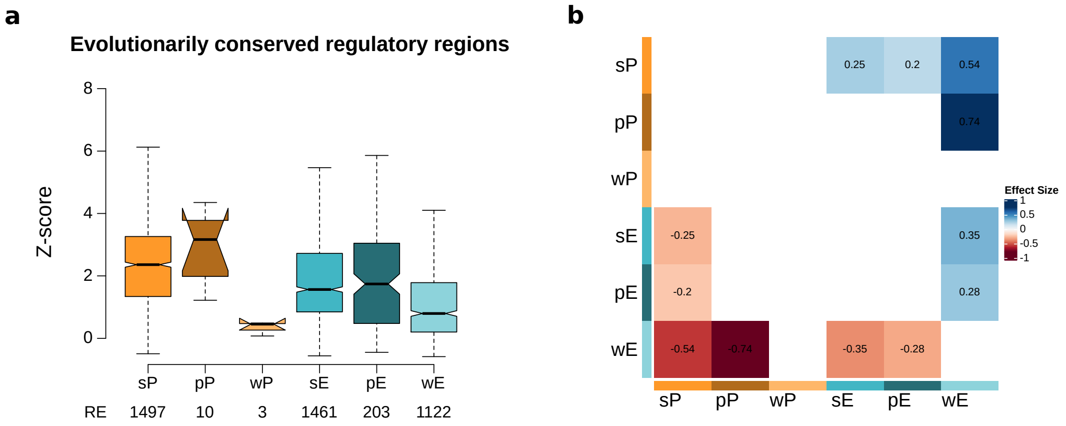

Supplementary Figure 16. Fully conserved orthologous regulatory elements with different epigenetic states show distinctive evolutionary dynamics. a, Distribution of *Z*-score values of evolutionarily conserved regulatory regions associated with human protein-coding genes (Kruskal-Wallis test, $P = 6.2 \times 10^{-124}$). Box plots show medians and the first and third quartiles (the 25th and 75th percentiles), respectively. The upper and lower whiskers extend the largest and smallest value no further than 1.5 × IQR. **b,** Effect sizes correspond to significant pairwise comparisons from (a) (Dwass-Steel-Critchlow-Fligner test). Only the effect sizes of significant comparisons are shown ($P < 0.05$).

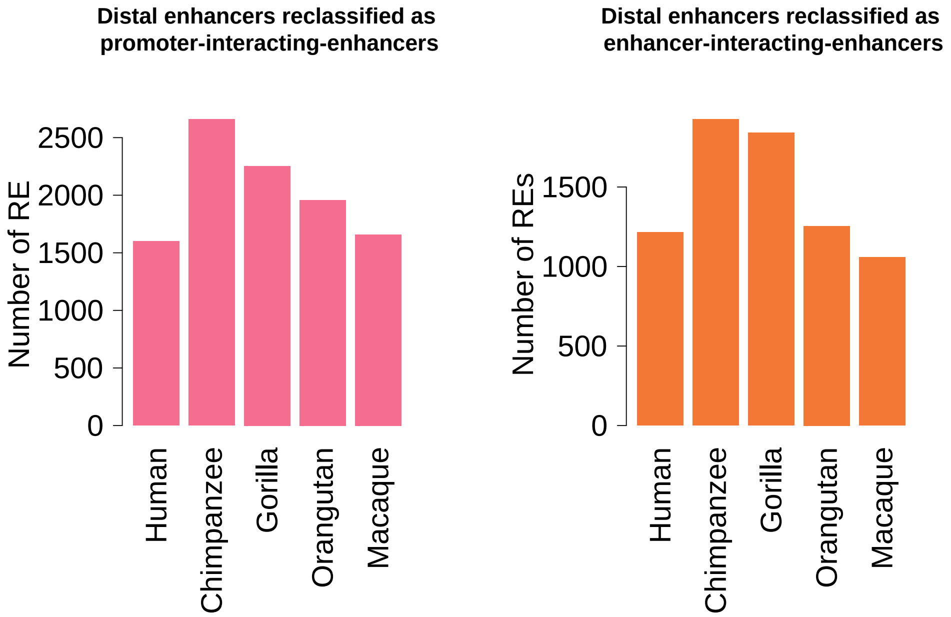

Supplementary Figure 17. Number of distal enhancers annotated as PiE and EiE per species through the integration of 3D chromatin data. Promoter-interacting-enhancers are gene-associated enhancers that interact with genic promoters. Enhancer-interacting-enhancers are gene-associated enhancers that interact with other enhancers.

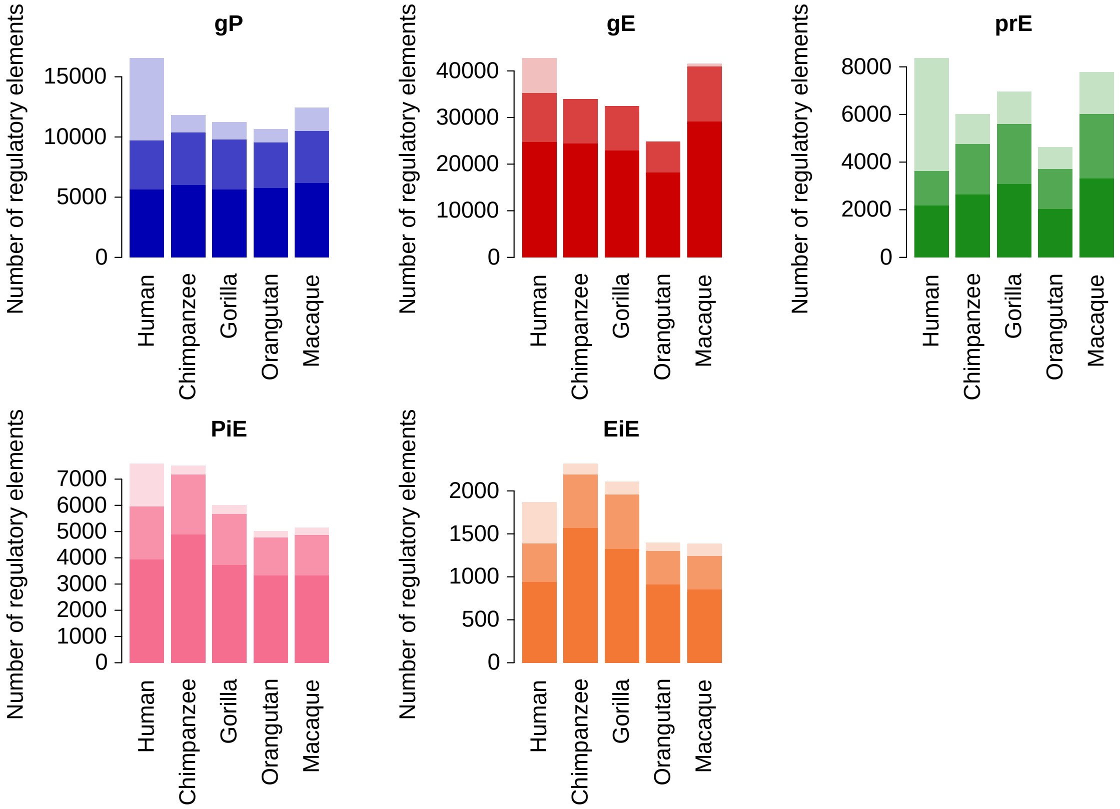

Supplementary Figure 18. Number of regulatory elements annotated as genic promoters (gP), intragenic enhancers (gE), proximal enhancers (prE), promoter-interacting enhancers (PiE) and enhancer-interacting enhancers (EiE). Dark, medium and light shades

indicate the number of regulatory elements associated with 1-to-1orthologous protein-coding genes, protein-coding genes or non-coding genes, respectively

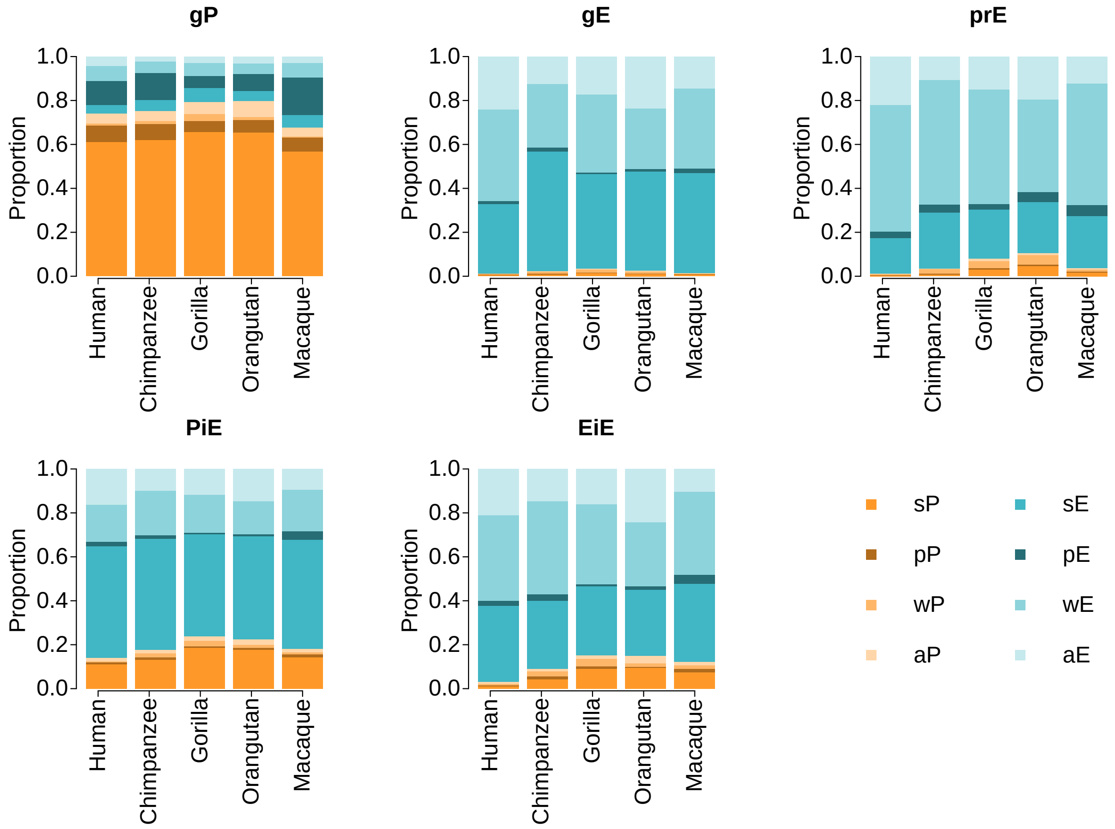

Supplementary Figure 19. Correspondence between regulatory state and component of regulatory elements associated with orthologous protein-coding genes. Each bar shows, for regulatory elements assigned a given regulatory component, the proportion of regulatory elements with the color-coded regulatory state. P: promoter; E: enhancer; s: strong; p: poised; w: weak; a: ambiguous (different activity states between biological replicates).

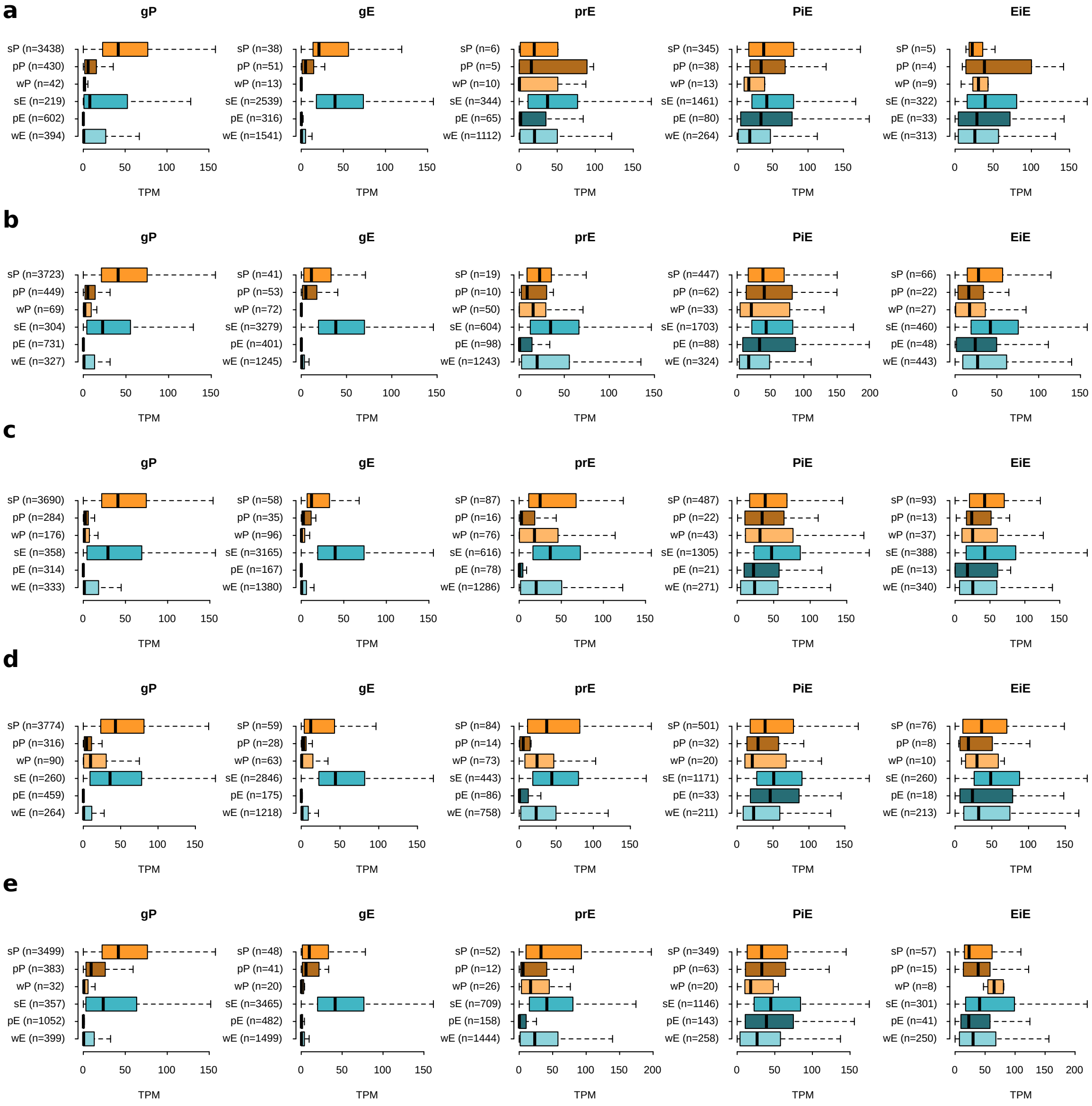

Supplementary Figure 20. Gene expression levels are associated with the regulatory state and the type of regulatory component of the associated regulatory elements. Box plots show, for every species and regulatory role, the distribution of the TPM expression levels of the genes they regulate. Distributions for **a,** human; **b,** chimpanzee; **c,** gorilla; **d,** orangutan and **e,** macaque. Box plots show medians and the first and third quartiles (the 25th and 75th percentiles), respectively. The upper and lower whiskers extend the largest and smallest value no further than 1.5 × IQR. Samples sizes are indicated in the plot. (Kruskal-Wallis test; $P_{Human\text{-}gP} = 0$; $P_{Human\text{-}gE} = 0$; $P_{Human\text{-}prE} = 2.2 \times 10^{-12}$; $P_{Human\text{-}PiE} = 6.3 \times 10^{-20}$; $P_{Human\text{-}EiE} = 0.0016$; $P_{Chimpanzee\text{-}gP} = 0$; $P_{Chimpanzee\text{-}gE} = 0$; $P_{Chimpanzee\text{-}prE} = 1.2 \times 10^{-27}$; $P_{Chimpanzee\text{-}PiE} = 1.4 \times 10^{-27}$; $P_{Chimpanzee\text{-}EiE} = 2.1 \times 10^{-08}$; $P_{Gorilla\text{-}gP} = 0$; $P_{Gorilla\text{-}gE} = 0$; $P_{Gorilla\text{-}prE} = 4.03 \times 10^{-34}$; $P_{Gorilla\text{-}PiE} = 5.9 \times 10^{-14}$; $P_{Gorilla\text{-}EiE} = 2.4 \times 10^{-05}$; $P_{Orangutan\text{-}gP} = 0$; $P_{Orangutan\text{-}gE} = 0$; $P_{Orangutan\text{-}prE} = 1.3 \times 10^{-30}$; $P_{Orangutan\text{-}PiE} = 1.4 \times 10^{-14}$; $P_{Orangutan\text{-}EiE} = 0.000996$; $P_{Macaque\text{-}gP} = 0$; $P_{Macaque\text{-}gE} = 0$; $P_{Macaque\text{-}prE} = 7.4 \times 10^{-42}$; $P_{Macaque\text{-}PiE} = 3.2 \times 10^{-14}$; $P_{Macaque\text{-}EiE} = 0.0056$)

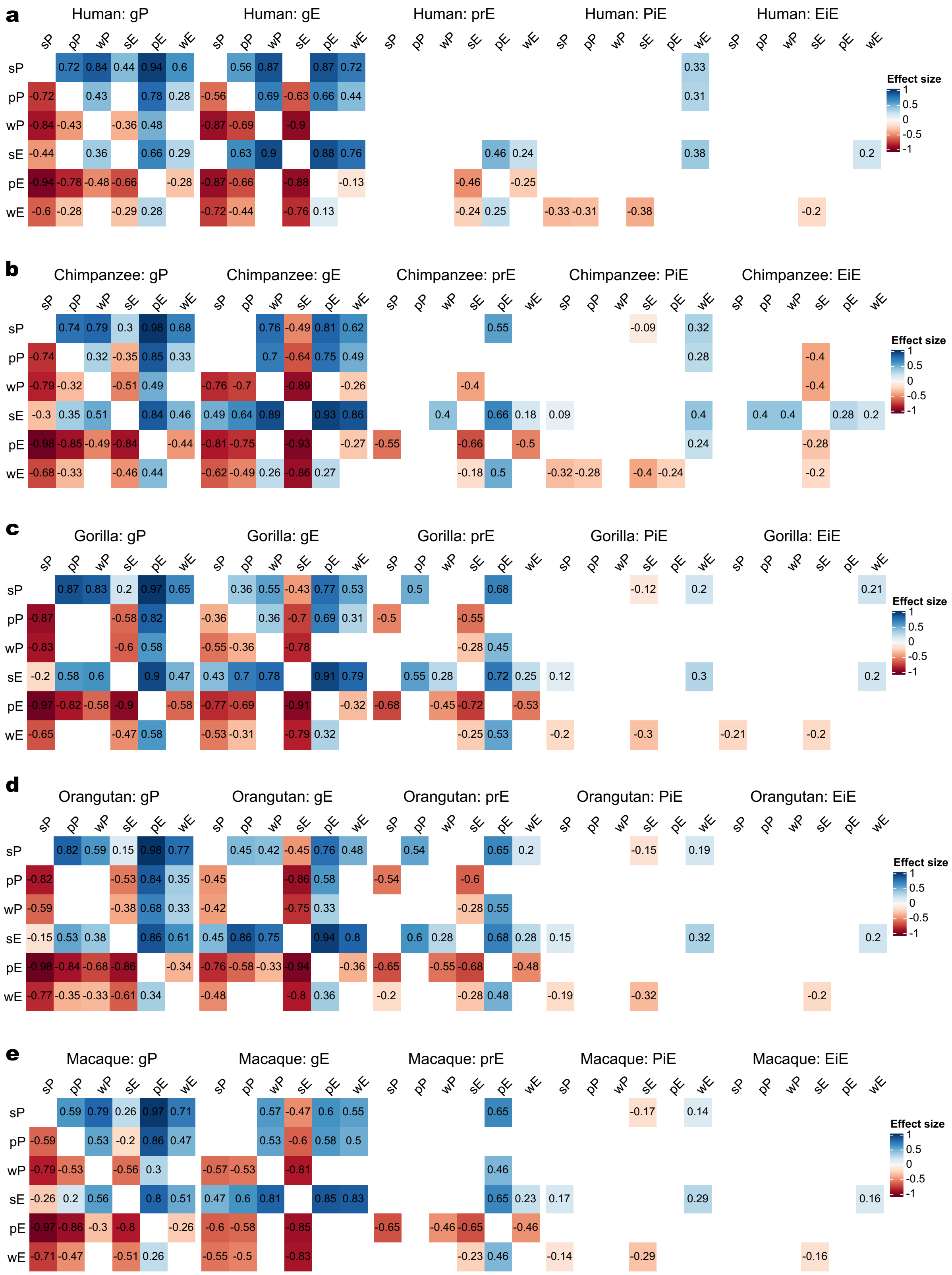

Supplementary Figure 21. The expression levels of genes associated with regulatory elements with strong activities are significantly higher than those associated with poised activities, which have lower expression levels. For each species and regulatory component, we compared the expression levels of the associated genes based on the presence of regulatory

elements with strong, poised or weak regulatory promoter or enhancer states (Methods). Cell values show the effect size for those significantly different pairwise comparisons (Dwass-Steel-Critchlow-Fligner test; $P < 0.05$) in **a,** human; **b,** chimpanzee; **c,** gorilla; **d,** orangutan and **e,** macaque.

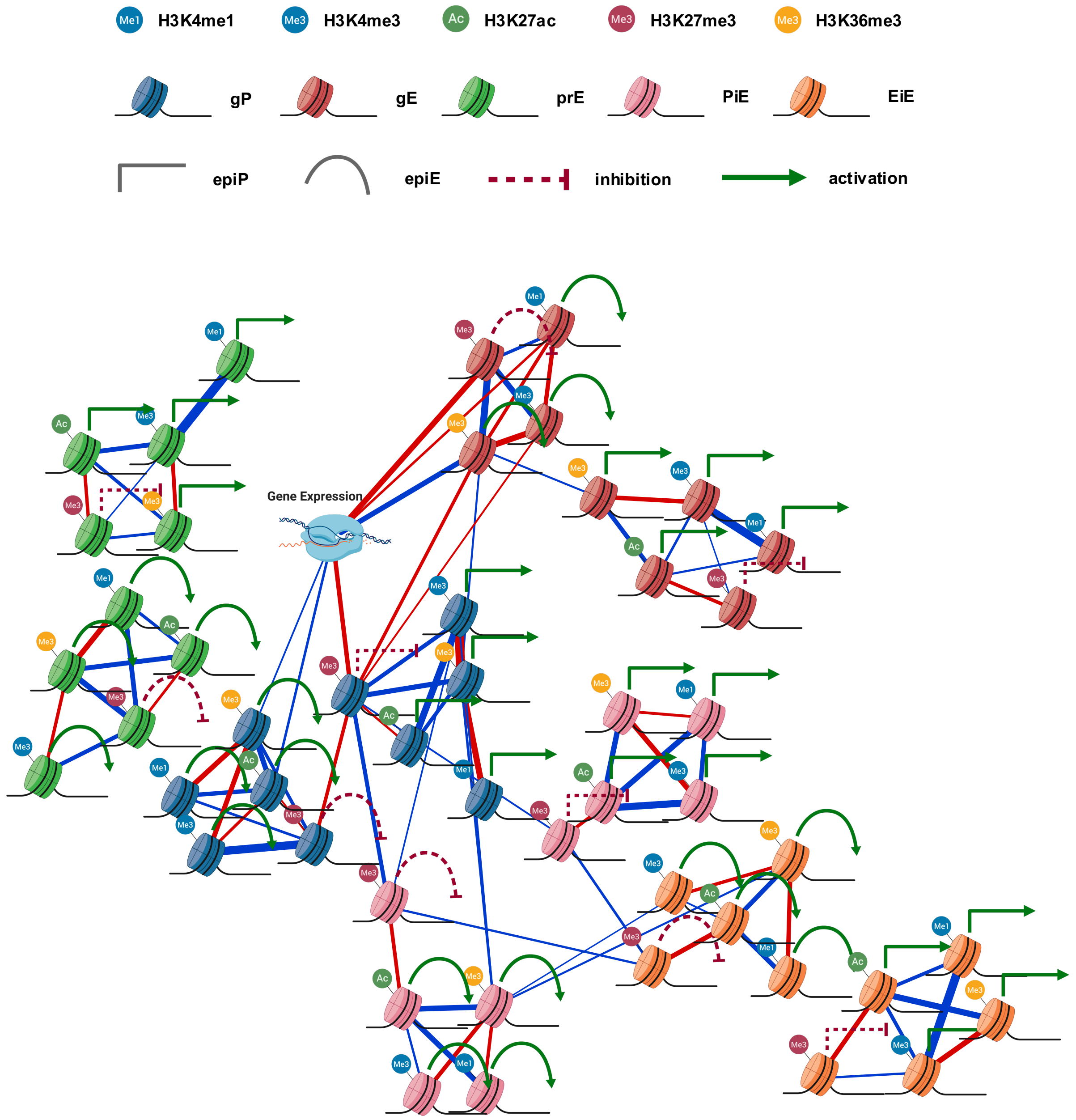

Supplementary Figure 22. Global residuals Sparse Partial Correlation Network. Network constructed using the residuals from linear models of the original variables and the first eigencomponent of each regulatory component (Methods). Blue edges represent positive partial correlations and red edges negative ones. Edge widths are proportional to absolute partial correlation values within each network. Only nodes for values with significant and relevant partial correlations were represented (minimal partial correlation = -0.323; maximal partial correlation = 0.354; all partial correlations Benjamini-Hochberg's *Q-value* $< 3.4 \times 10^{-112}$).

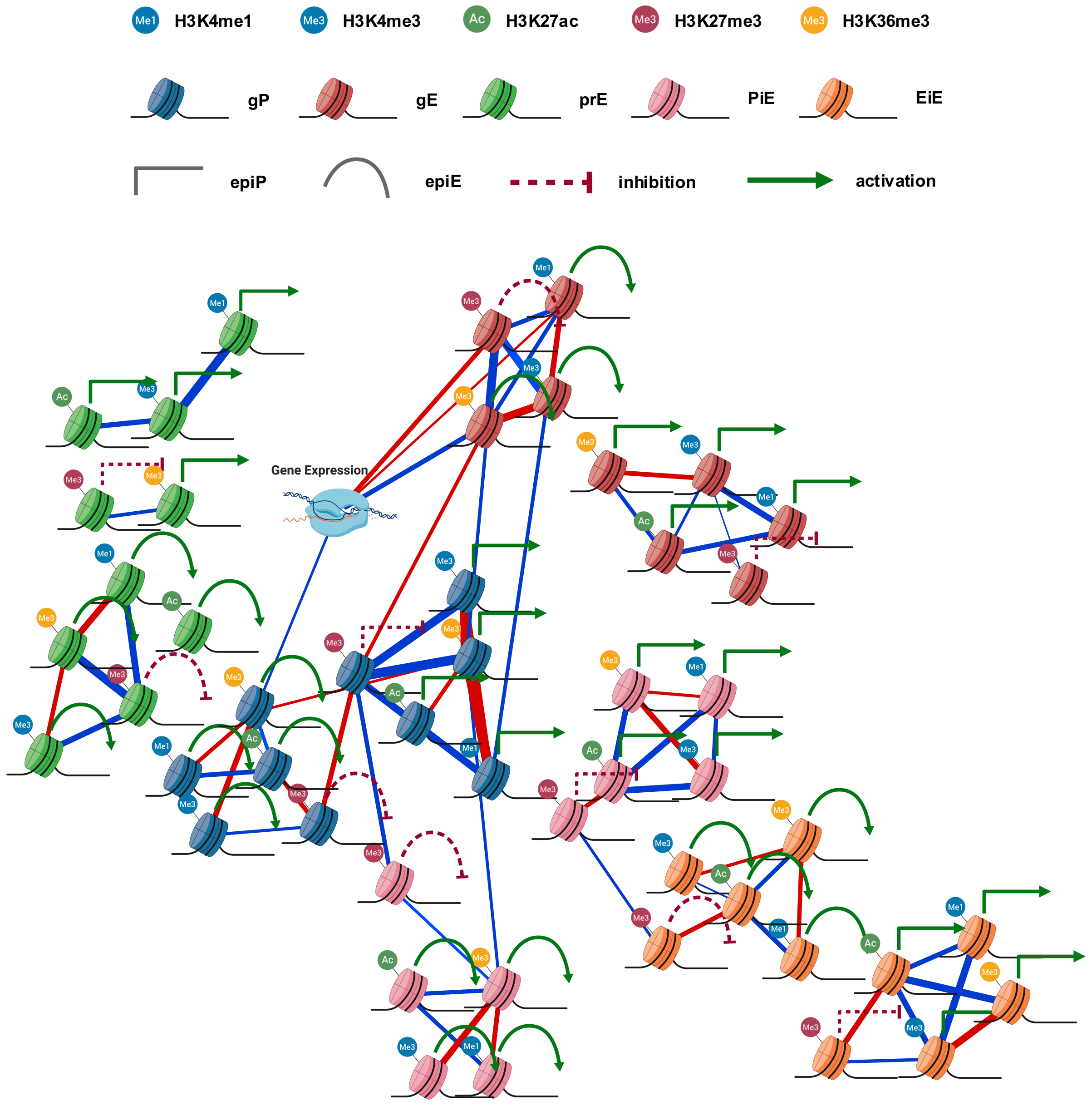

Supplementary Figure 23. Global residuals Sparse Partial Correlation Network for genes with a full architecture. Network constructed using the residuals from linear models of the original variables and the first eigencomponent of each regulatory component (Methods) considering only genes with a full architecture (genes with at least one element in every type of component; n = 1,068 genes). Blue edges represent positive partial correlations and red edges negative ones. Edge widths are proportional to absolute partial correlation values within each network. Only nodes for values with significant and relevant partial correlations were represented (minimal partial correlation = -0.581; maximal partial correlation = 0.448; all partial correlations Benjamini-Hochberg's *Q-value* < 3.3 x 10^{-97}).

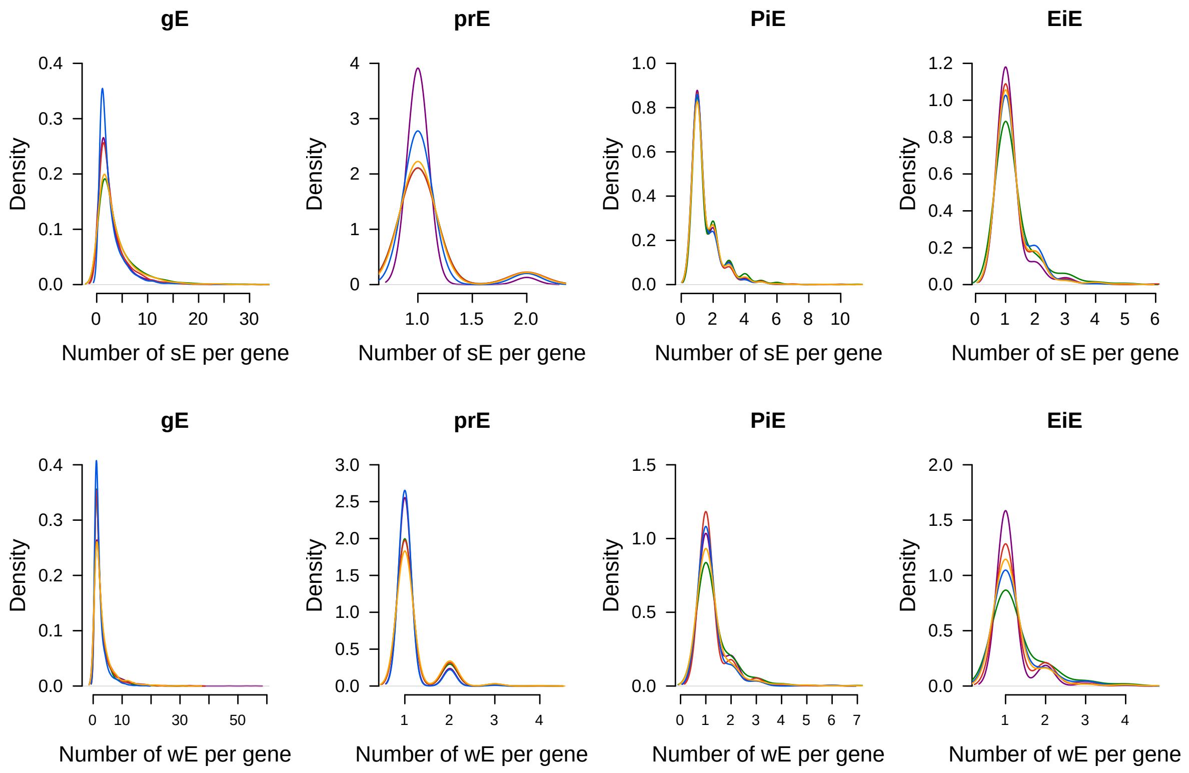

Supplementary Figure 24. Distribution of the number of regulatory elements with strong and weak enhancers states associated with a gene, stratified by their regulatory component. a, Number of sE in each regulatory component. **b,** Number of wE in each regulatory component. Species are color-coded: human (purple), chimpanzee (green), gorilla (red), orangutan (blue) and macaque (yellow).

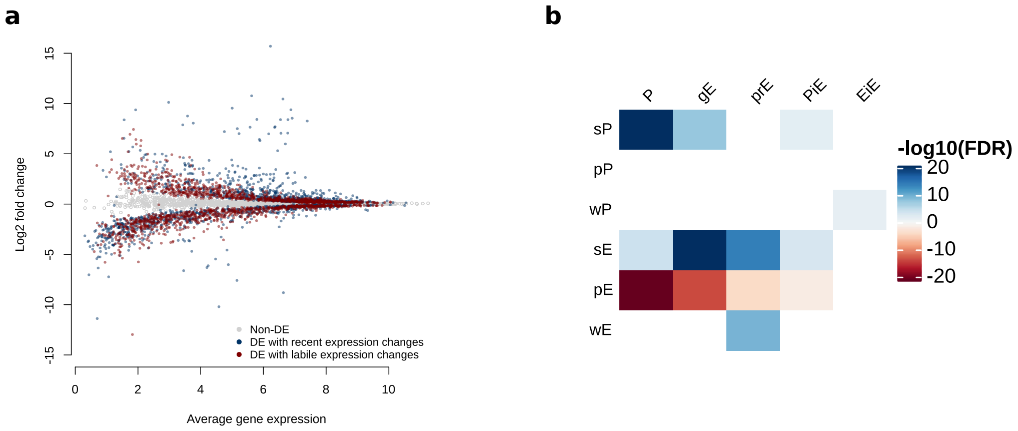

Supplementary Figure 25. Expression differences between species. a, Genes with expression changes between species. MA plot showing the average gene expression versus the expression change (log2 fold change) for genes non-differentially expressed between species (grey), genes with species-specific expression changes (blue) and genes with non-species-specific expression changes (red). **b,** Gene expression differences between species are significantly associated with changes in the number of regulatory elements with strong and poised activities, mainly at genic promoters and intragenic enhancers. Cell values in the heatmap show the multiple testing adjusted P-value from a one-tailed Wilcoxon signed-rank test testing whether a higher number of regulatory elements in the corresponding component-state combinations is significantly associated with higher (blue) or lower (red) expression levels in genes with expression differences across species.

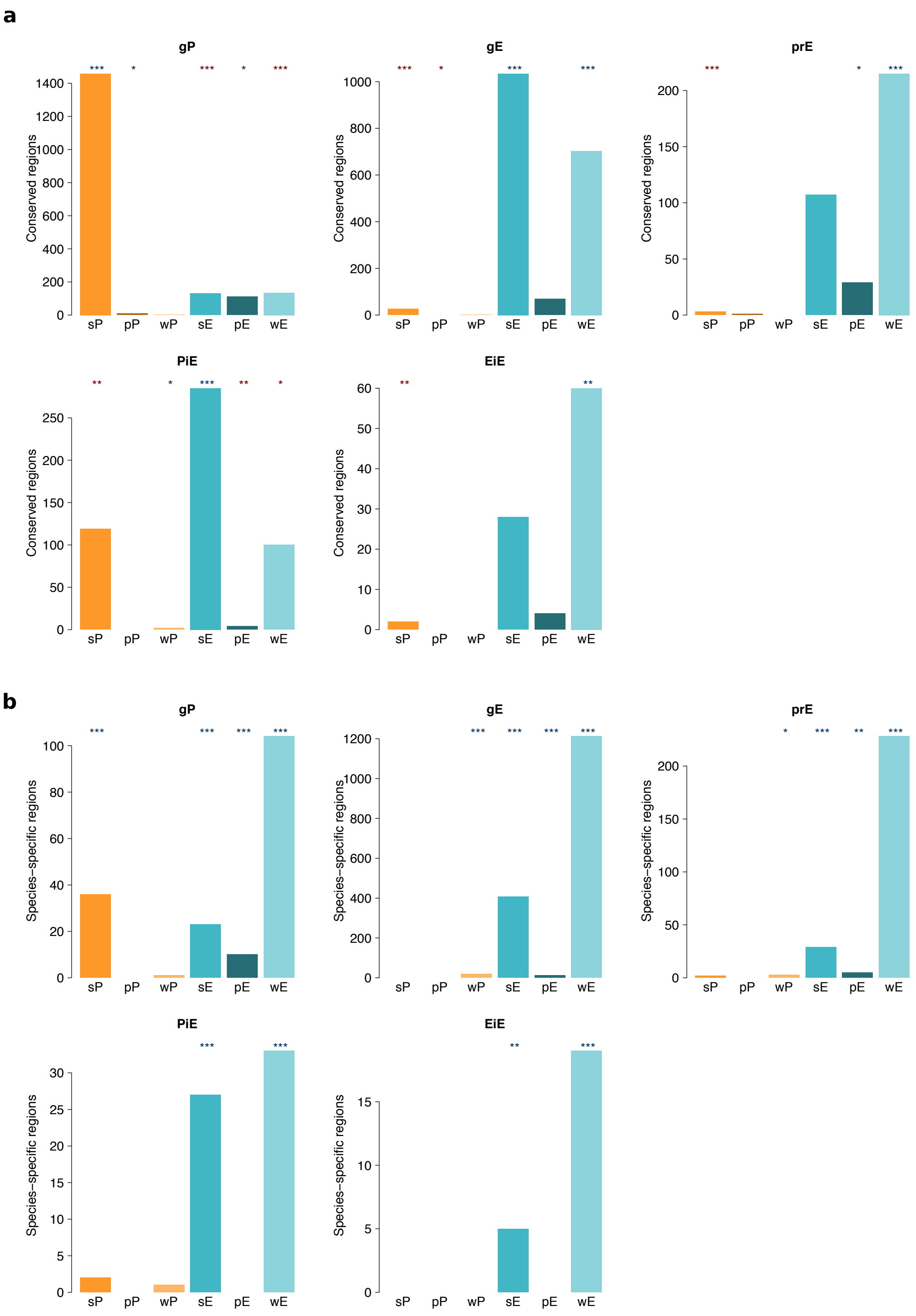

Supplementary Figure 26. Evolutionarily conserved and species-specific regulatory states are enriched in particular regulatory components. For each type of component, bar plots show the number of **a,** fully evolutionarily conserved or **b,** species-specific orthologous regulatory regions with the corresponding color-coded regulatory state fully conserved. Asterisks represent the magnitude of a Chi-square test residuals, where blue and red indicate there are either more or fewer elements, respectively, in that particular component-state combination than expected by random chance (* abs(residuals) > 2; ** abs(residuals) > 4; *** abs(residuals) > 8; Chi-square test; $P = 0.0004998$).

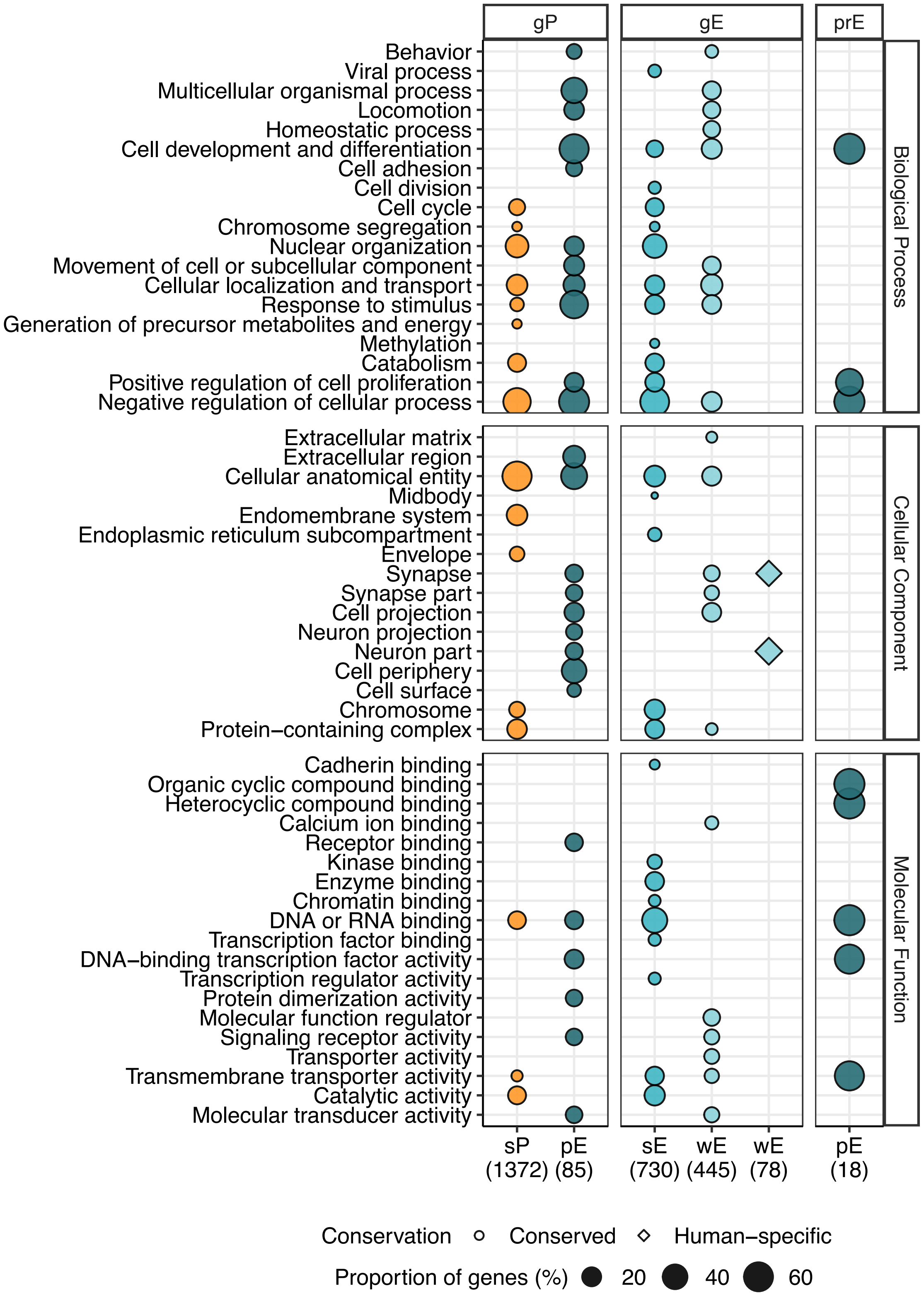

Supplementary Figure 27. Functional enrichment of genes associated with fully conserved and species-specific epigenetic state/component combinations. Functional enrichment of significantly enriched groups in Supplementary Fig. 26. The size of circles/diamonds indicates the proportion of genes included in each functional category out of the total number of genes contained in the corresponding regulatory group (shown in brackets). A simplified version of this figure that only includes Biological Process and Cellular Component categories for genic promoter and intragenic enhancer combinations is shown in Fig. 5a.

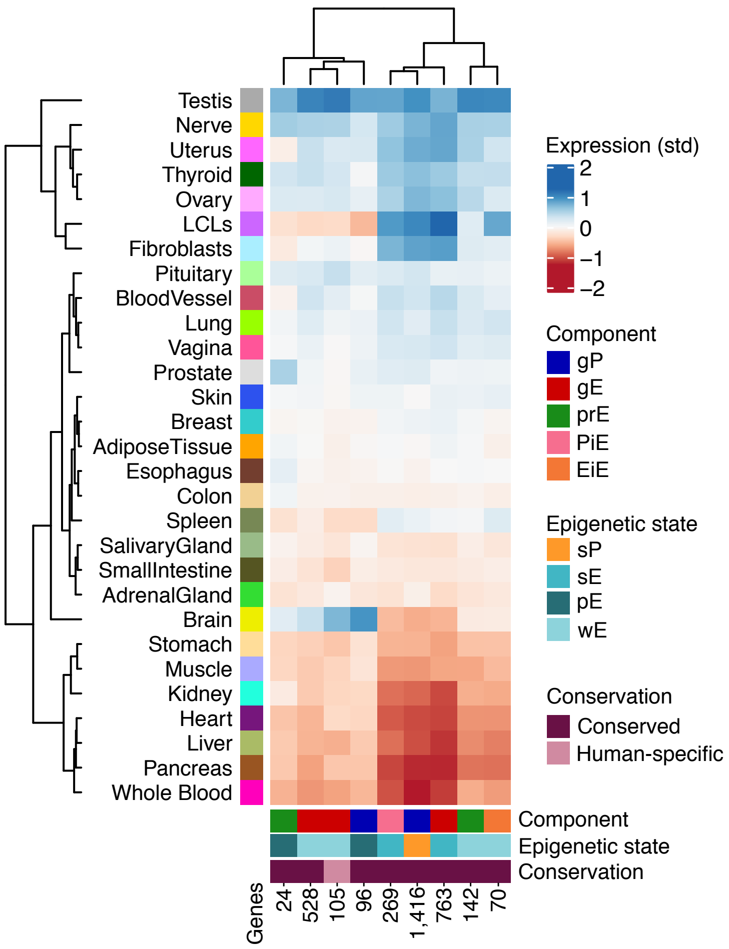

Supplementary Figure 28. Heatmap of standardized expression of genes associated with fully conserved and species-specific epigenetic state/component combinations. Epigenetic state/component combinations of enriched groups in Supplementary Fig. 26. Standardized tissue expression from bulk RNA-seq data were obtained from GTEx (v8). A simplified version of this figure, including combinations with significant functional enrichments, is shown in Fig. 5b.

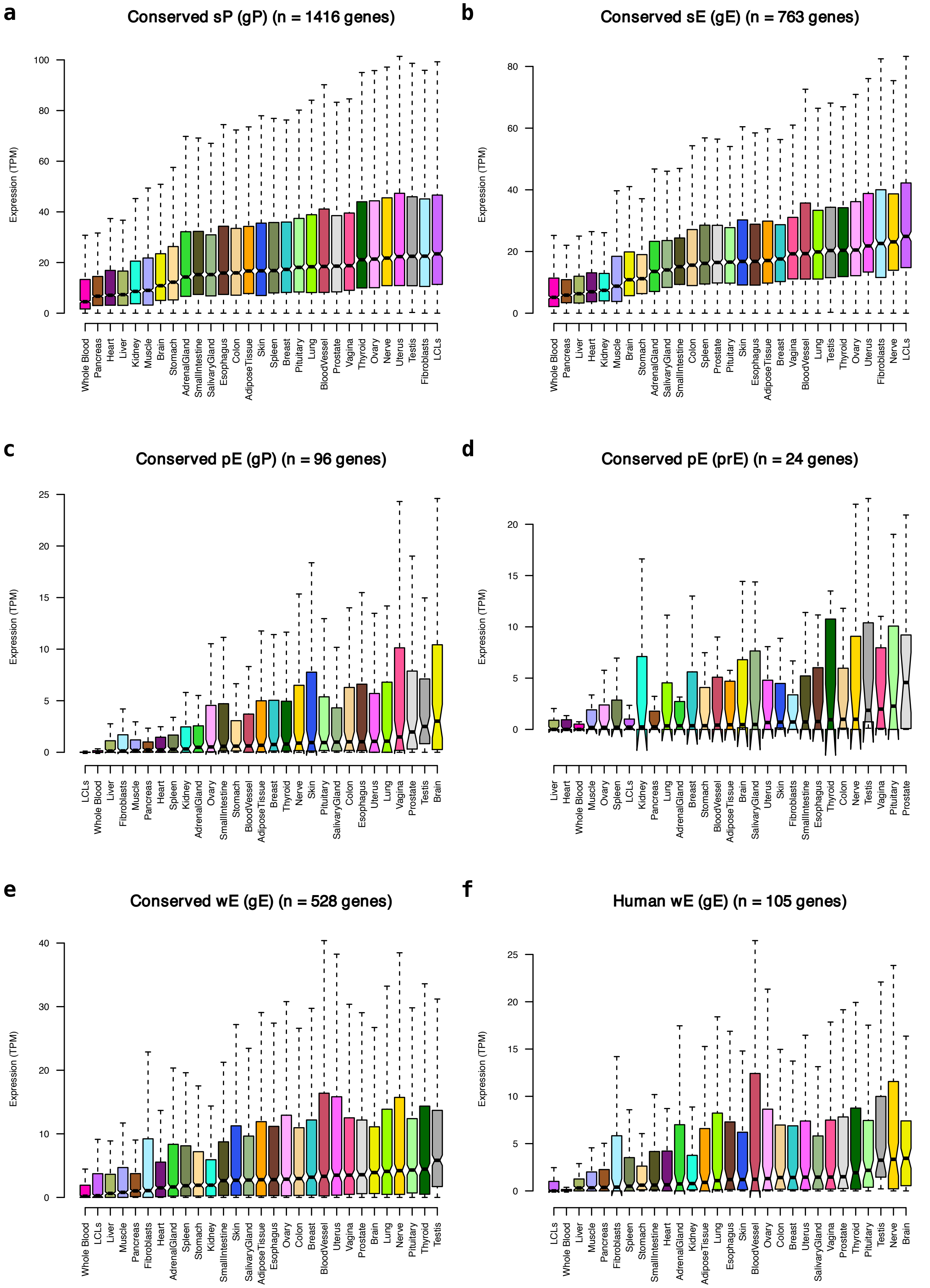

Supplementary Figure 29. Tissue expression of genes associated with particular conserved or human-specific component-state combinations with functional enrichments. Tissue expression (median TPM) from bulk RNA-seq data obtained from the latest GTEx release (v8) in genes that have

regulatory elements that are conserved **a,** genic promoters with strong promoter activities; **b,** intragenic enhancers with strong enhancer activities; **c,** genic promoters with poised enhancer activities; **d,** proximal enhancers with poised enhancer activities; **e,** intragenic enhancers with weak enhancer activities and **f,** human-specific intragenic enhancers with weak enhancer activities (hswEgE). Box plots show medians and the first and third quartiles (the 25th and 75th percentiles), respectively. The upper and lower whiskers extend the largest and smallest value no further than 1.5 × IQR.

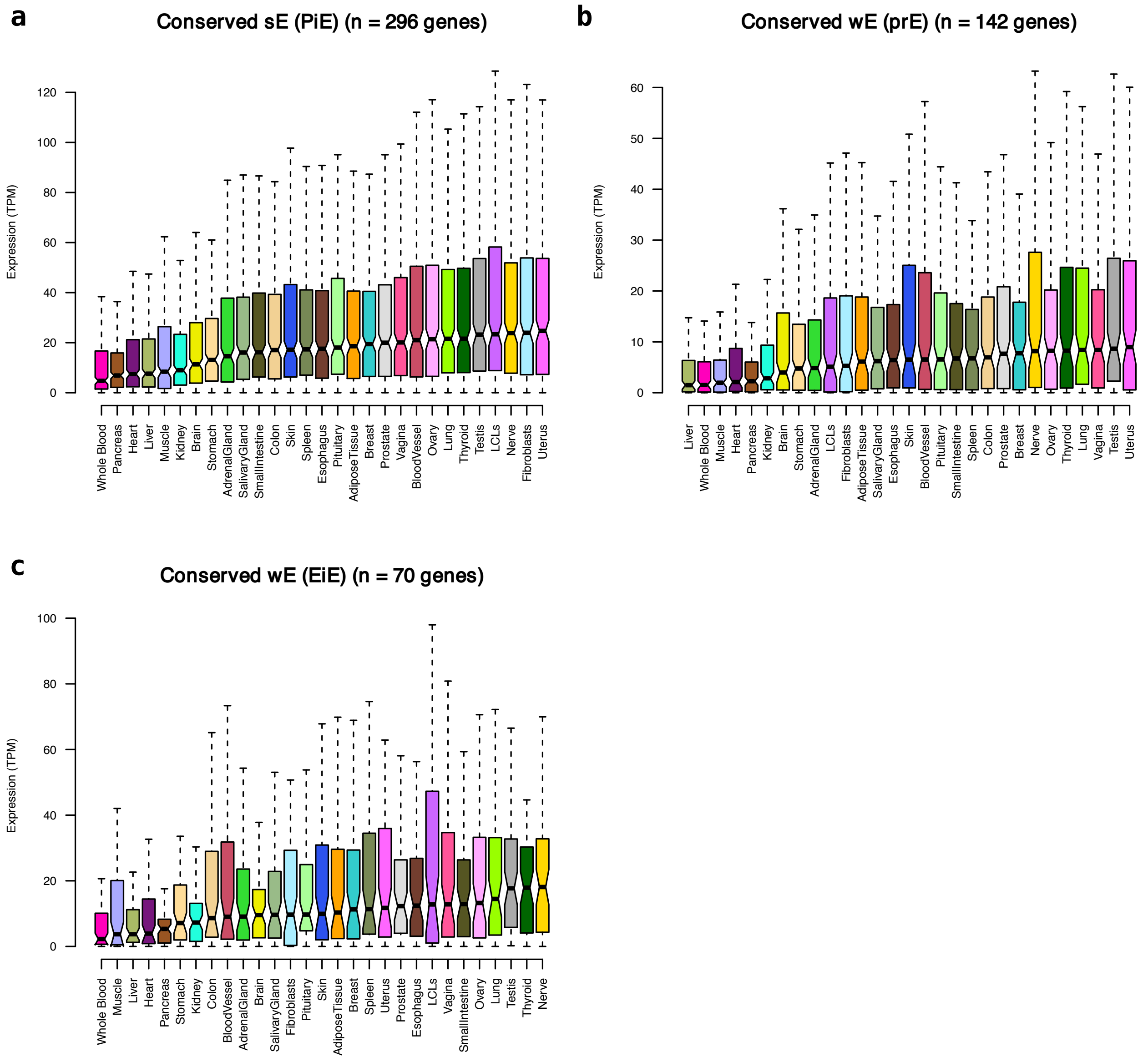

Supplementary Figure 30. Tissue expression of genes associated with particular conserved or human-specific component-state combinations without significant functional enrichments. Tissue expression patterns (median TPM) from bulk RNA-seq data obtained from the latest GTEx release (v8) in genes that have conserved regulatory elements that are **a,** promoter-interacting enhancers with strong enhancer activities; **b,** proximal enhancers with weak enhancer activities and **c,** enhancer-interacting enhancers with weak enhancer activities. Box plots show medians and the first and third quartiles (the 25th and 75th percentiles), respectively. The upper and lower whiskers extend the largest and smallest value no further than 1.5 × IQR.

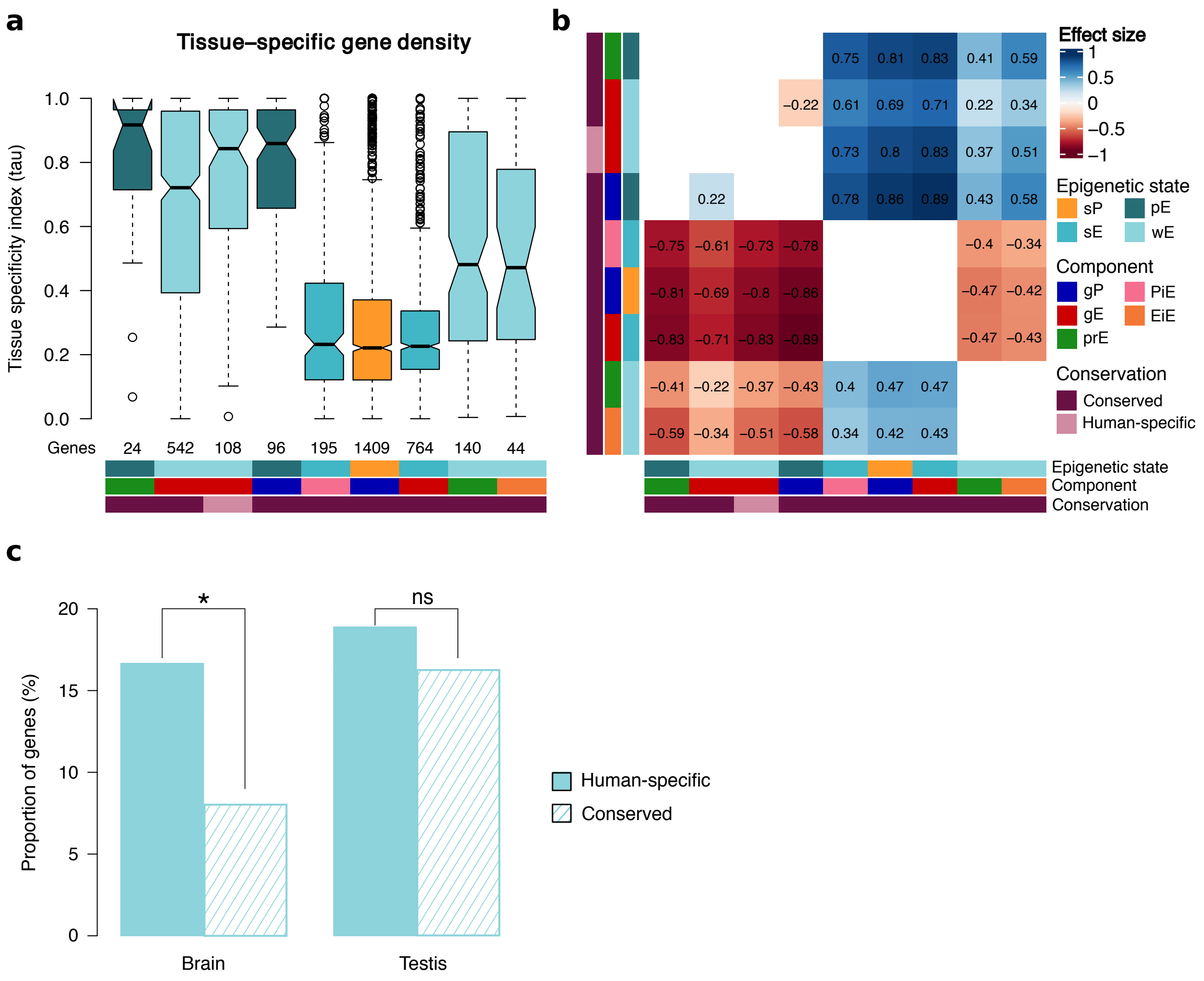

Supplementary Figure 31. Tissue specificity analyses show different patterns across regulatory components and epigenetic states. a, Distributions of the tissue-specific indices (tau) for genes associated with particular component-state combinations. Box plots show medians and the first and third quartiles (the 25th and 75th percentiles), respectively. The upper and lower whiskers extend the largest and smallest value no further than 1.5 × IQR. **b,** Effect sizes corresponding to significant pairwise comparison from (a) (Dwass-Steel-Critchlow-Fligner test; $P < 0.05$). **c,** Comparison of the proportion of brain-specific ($tau_{Brain} > 0.8$) and testis-specific genes ($tau_{Testis} > 0.8$) associated with either human-specific (n = 90 genes) or conserved intragenic enhancers with weak enhancer states (n = 524 genes). Two-tailed Fisher's exact test; P-values below and above 0.05 are indicated by an asterisk (*) or ns, respectively.

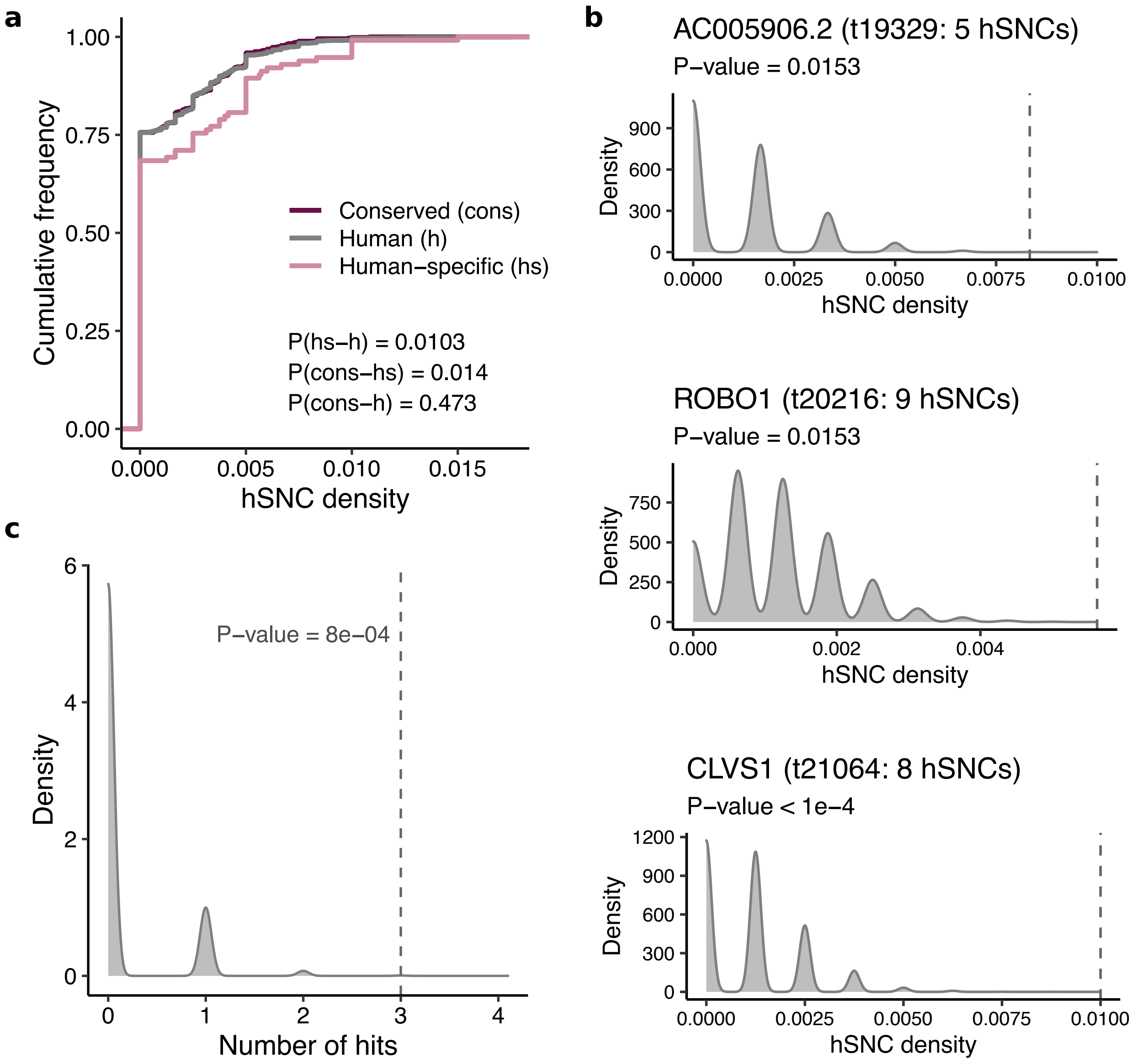

Supplementary Figure 32. Human-specific nucleotide changes (hSNC) in hswEgE. a, Distribution of hSNC densities in human genes associated with intragenic enhancers (grey), in the subset of genes associated with fully conserved weak intragenic enhancers (purple) and in the subset of genes with human-specific weak intragenic enhancers (hswEgE, pink). P-values in the plot correspond to the indicated one-tail Mann-Whitney U tests. **b,** Three hswEgE that accumulate more hSNCs than expected. For each of the significant hits, the name of the hswEgE-containing gene, the enhancer id (t), the number of hSNCs in the enhancer and the P-value is shown. **c,** Significance of the observed number of hits. Distribution of the simulated number of hits after 10,000 simulations. In **b,** and **c,** the vertical dashed line represents the value of the observed density for each enhancer and the observed number of hits in our data, respectively, and P-values are adjusted using Bonferroni correction.

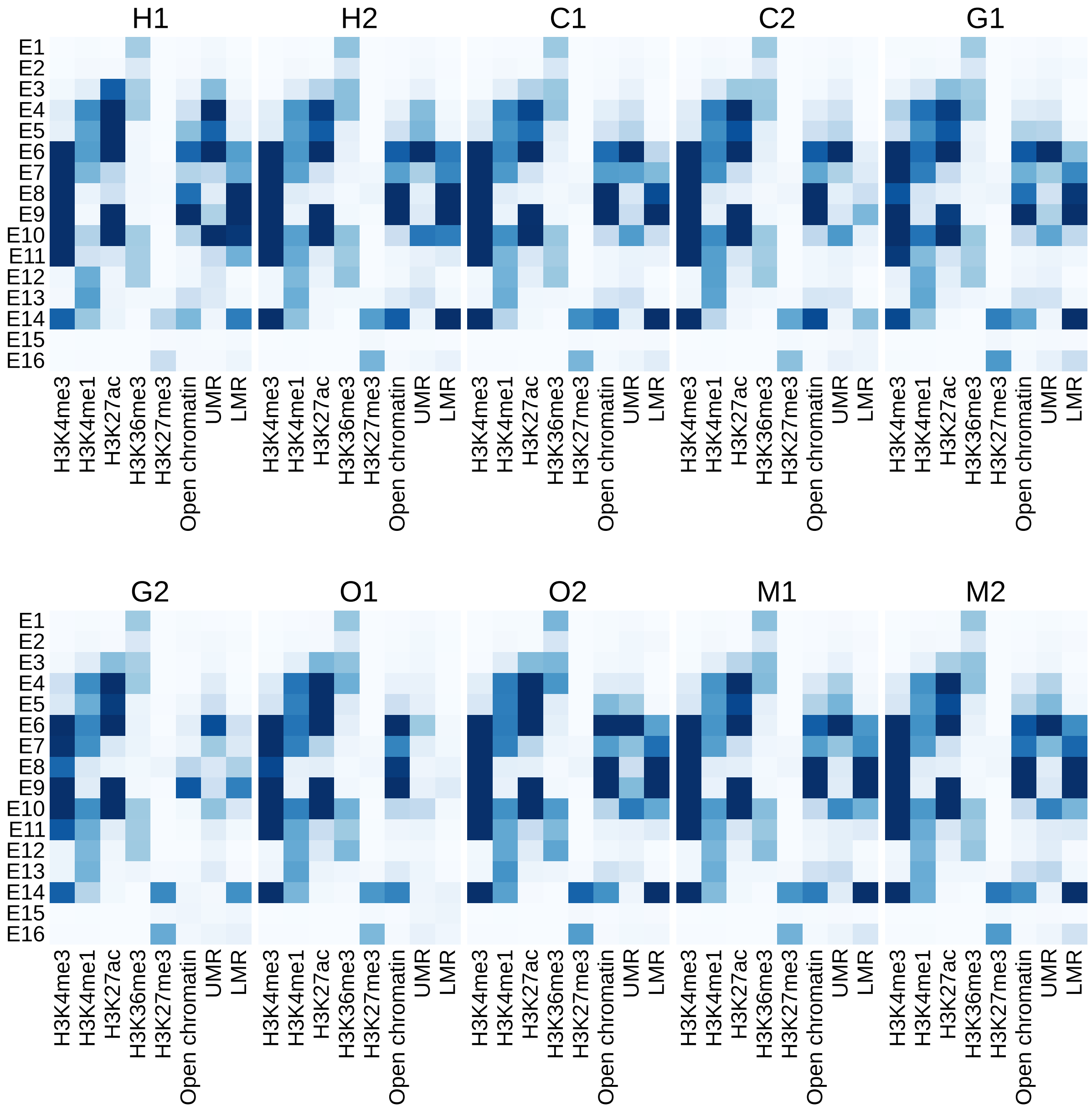

Supplementary Figure 33. Histone and open chromatin peaks are enriched in the expected chromatin states. See Supplementary Fig. 2.

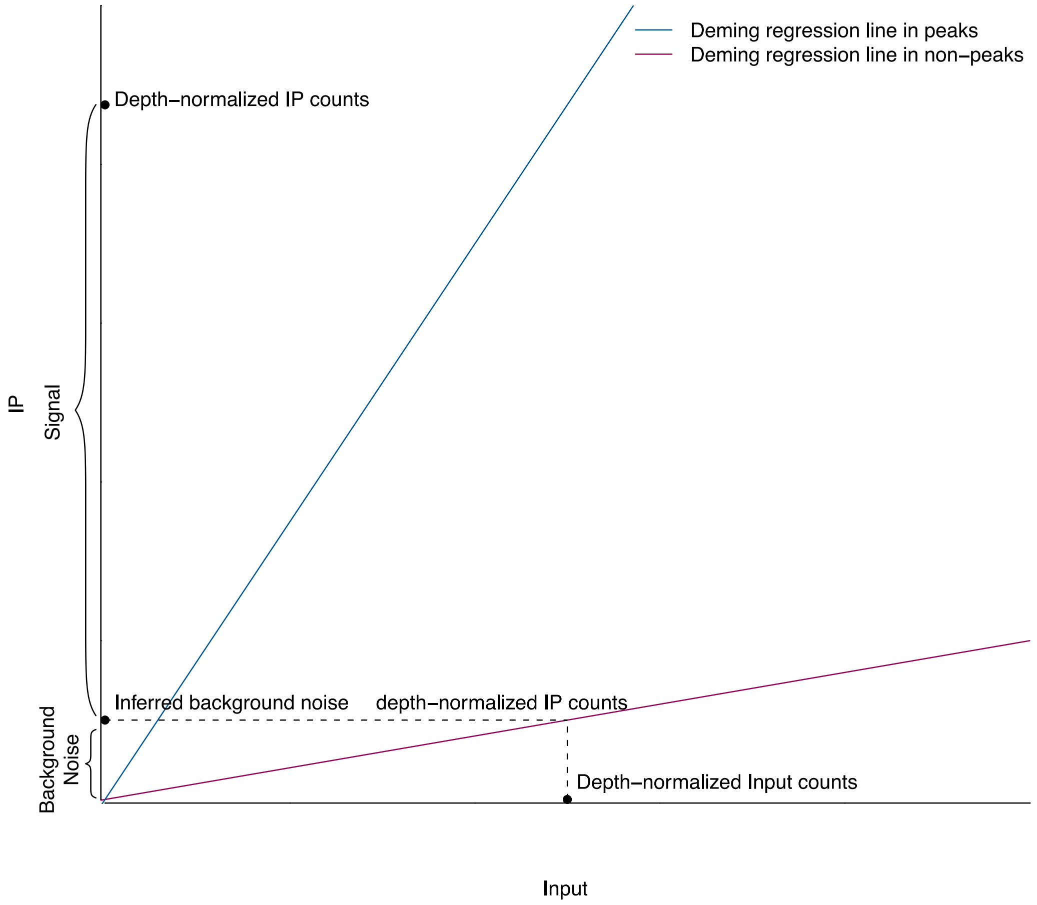

Supplementary Figure 34. Schematic representation of how background noise signal is removed from the immunoprecipitated (IP) signal. Regression parameters are estimated in a robust set of sample peaks and non-peaks and used later on to adjust the IP signal recovered at regulatory elements and orthologous regulatory regions (Supplementary Methods).

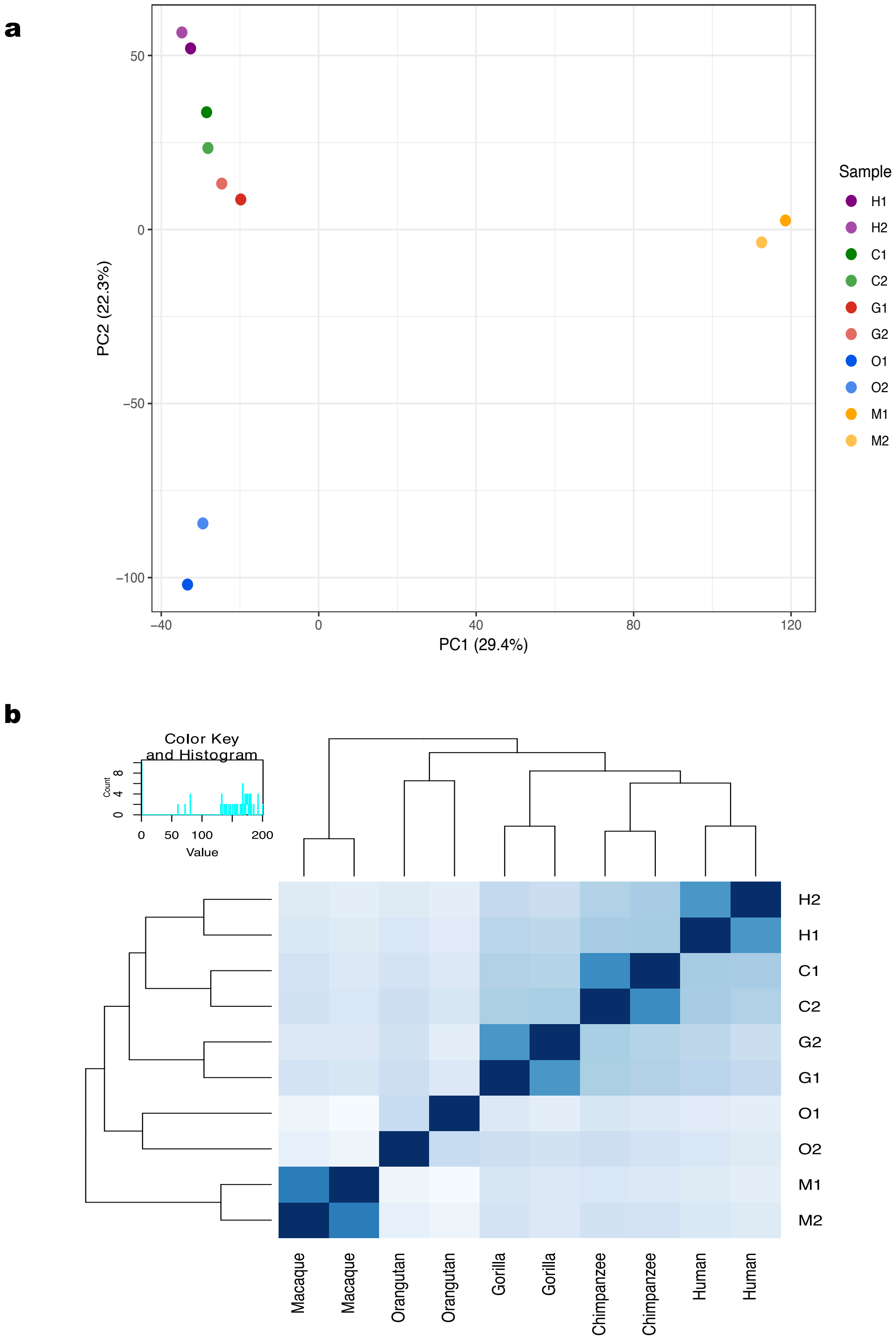

Supplementary Figure 35. Gene expression patterns recapitulate the known phylogenetic relationships between species. a, PCA and **b,** heatmap of the sample-to-sample Euclidean distances based on the expression levels of 1-to-1 orthologous protein-coding genes.

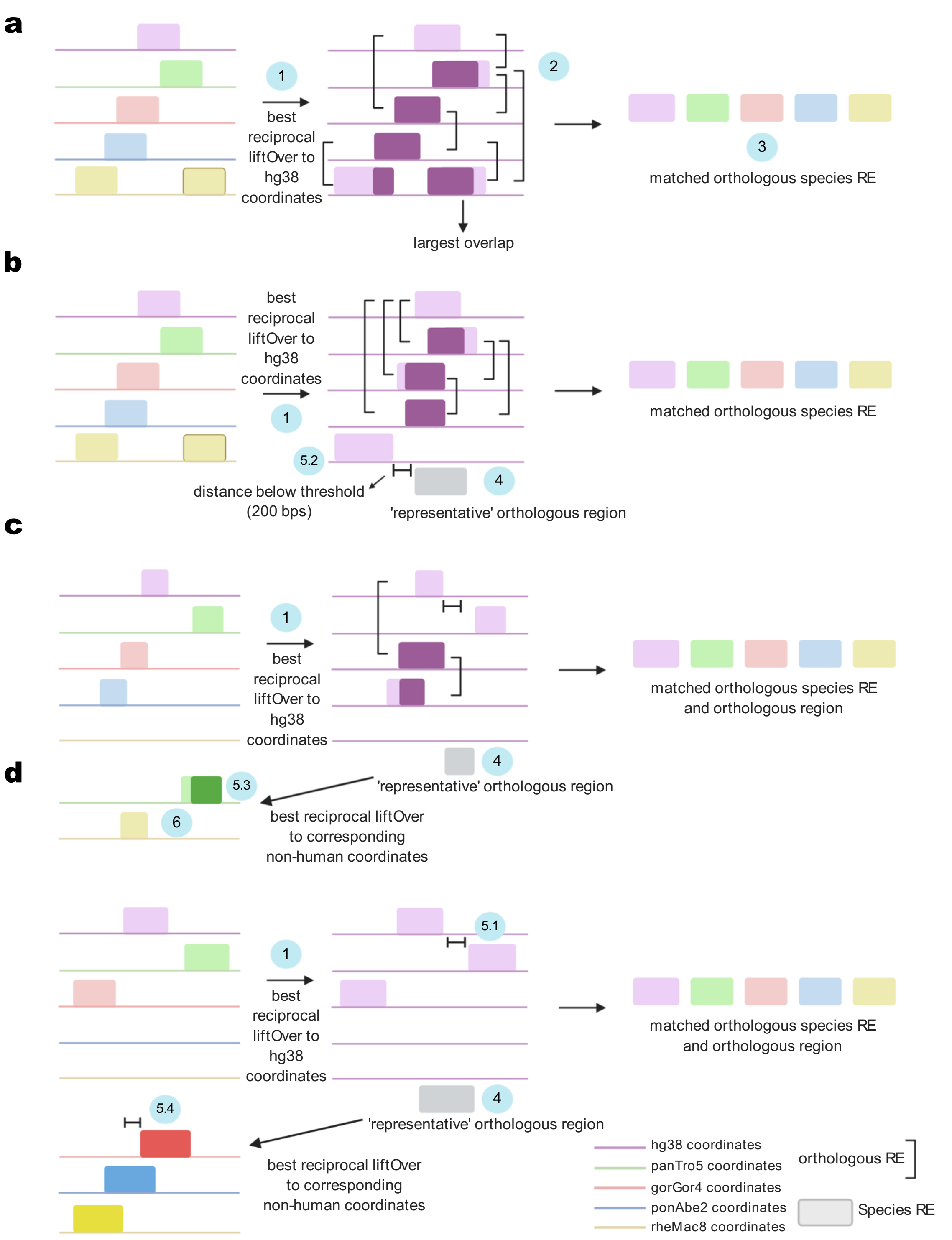

Supplementary Figure 36. Schematic representation of how orthologous relationships among species regulatory elements are established. Several different scenarios are illustrated. Boxes represent regulatory elements (RE). Colors represent the different reference genome coordinates (purple: human; green: chimpanzee; red: gorilla; blue: orangutan; yellow: macaque). Dark colors indicate overlapping bp. Outlined boxes with color gradient denote orthologous regions. Numbers in circles refer to the steps described in the text. **a,** Pairwise overlaps are found for all species. **b**, Although the macaque RE does not overlap with the REs of the other species, the representative orthologous region is in close proximity to the macaque

RE which is then recovered as the corresponding orthologous. **c,** Neither the chimpanzee nor macaque REs overlap with REs from the remaining species. When the representative orthologous region is mapped to the chimpanzee reference genome, a RE is found in close proximity and recovered as orthologous RE. When the representative orthologous region is mapped to the macaque reference genome, no RE is found, but the coordinates can be mapped and this genomic region is recovered as the corresponding orthologous region in macaque. In downstream quantitative analysis, reads will be counted and normalized in defined orthologous regions despite absence of RE. This approach allows the recovery of overlooked RE. **d,** Gorilla orthologous RE is recovered through proximity to the reference orthologous RE in gorilla coordinates. Orangutan and macaque are assigned their corresponding orthologous regions.

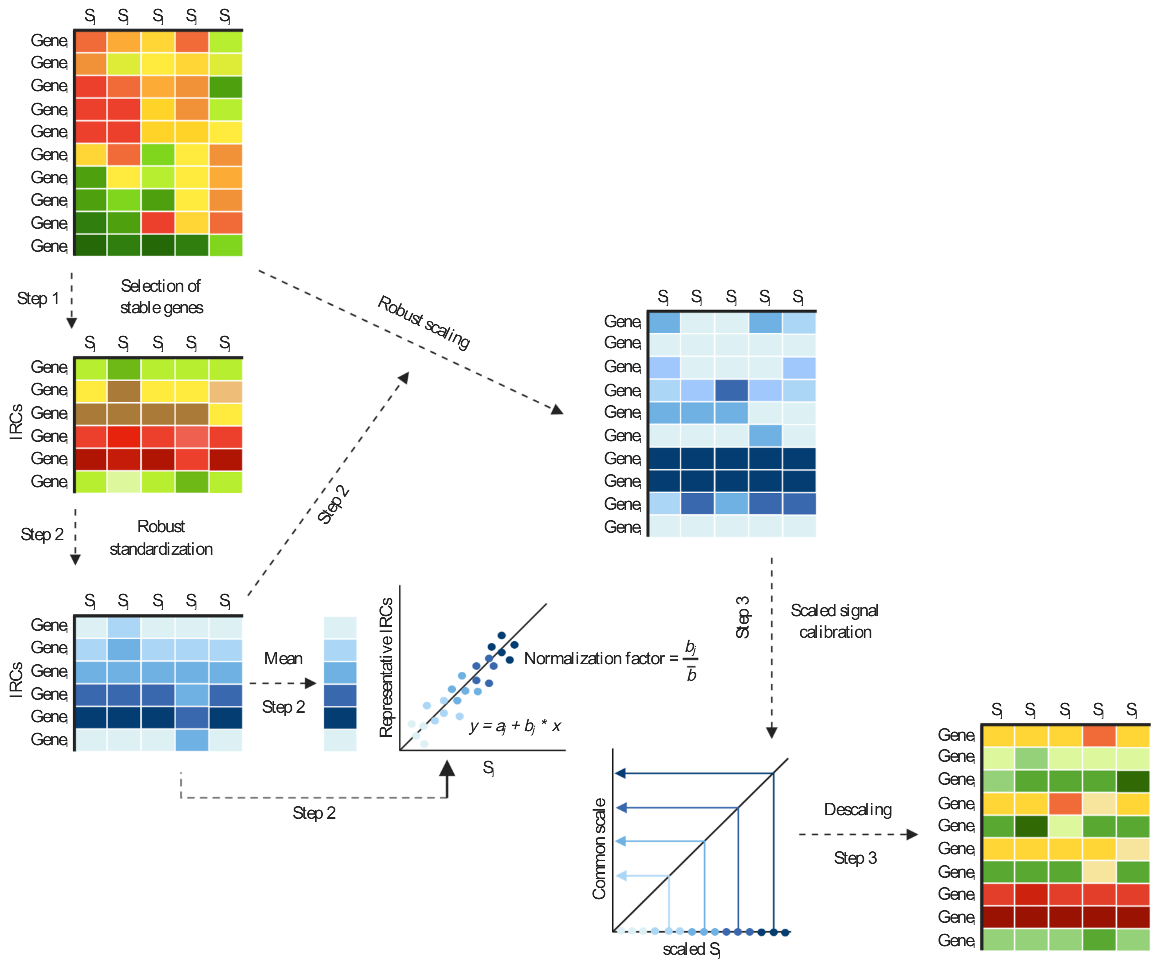

Supplementary Figure 37. Schematic representation of the approach employed to calibrate the expression and epigenetic signals between species. Step 1. Color gradient represents different degrees of expression/enrichments (green: low expression/enrichment; yellow: medium expression /enrichment; red: high expression/enrichment). IRCs are composed of units (genes, regulatory elements, epigenetic signals associated with a particular type of gene component) with similar enrichments across samples. **Step 2**. After robust standardization, values are normally distributed and more similar across samples (blue gradient; light blue: low enrichment; blue: medium enrichment; dark blue: high enrichment). Normalization factors are estimated considering the linear relationships between sample-specific and representative robust standardized values at IRCs. **Step 3**. Values at genes/elements/components are scaled to the common space where normalization factors are computed and after the calibration, values are brought back to the original scale. S stands for sample and j ∈ [1,10]. N is the number of observations and I ∈ [1,N].

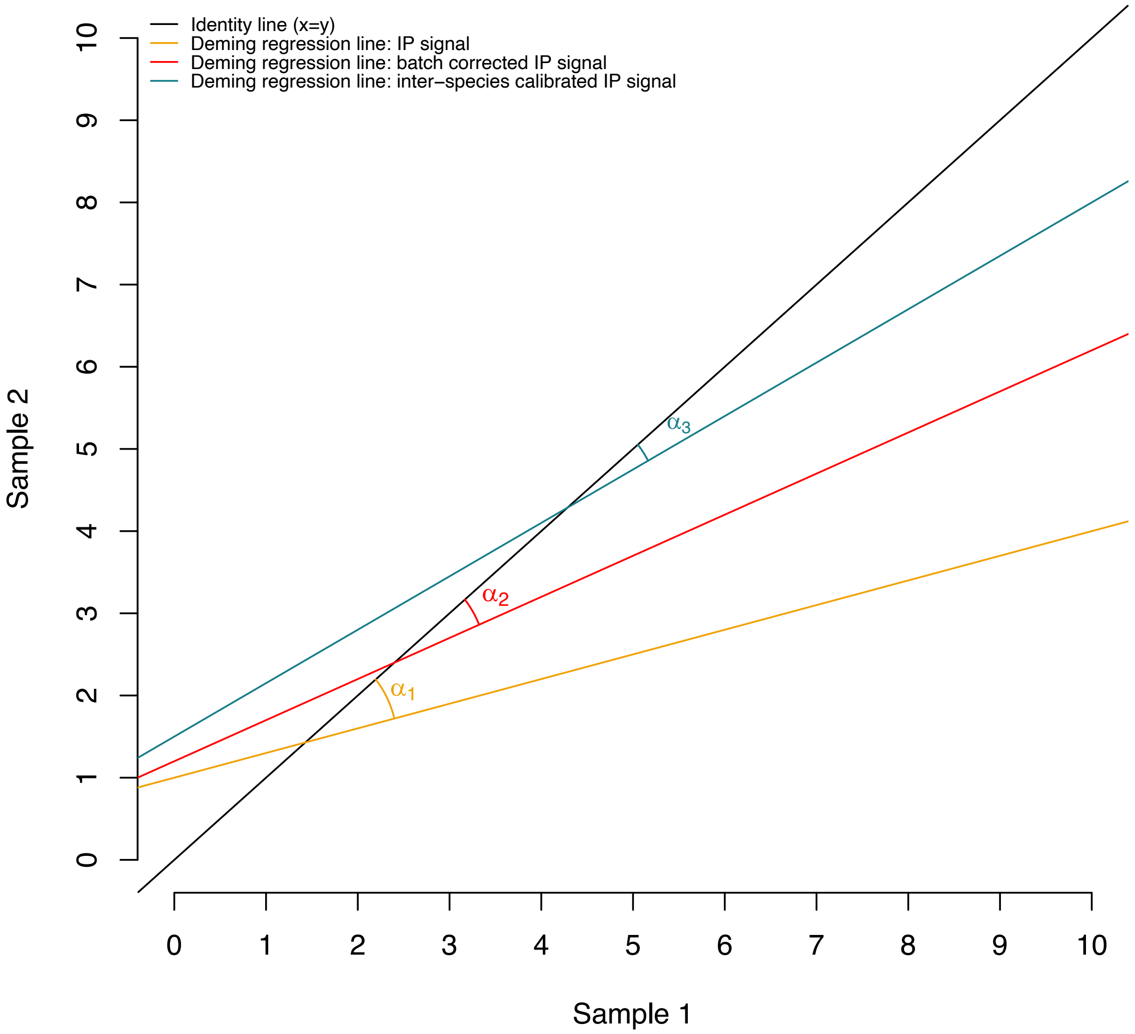

Supplementary Figure 38. Schematic representation of how the performance of our calibration method is evaluated. A progressive reduction in the angles to the identity line is expected upon successful normalization of the signal: $\alpha_1 < \alpha_2 < \alpha_3$.

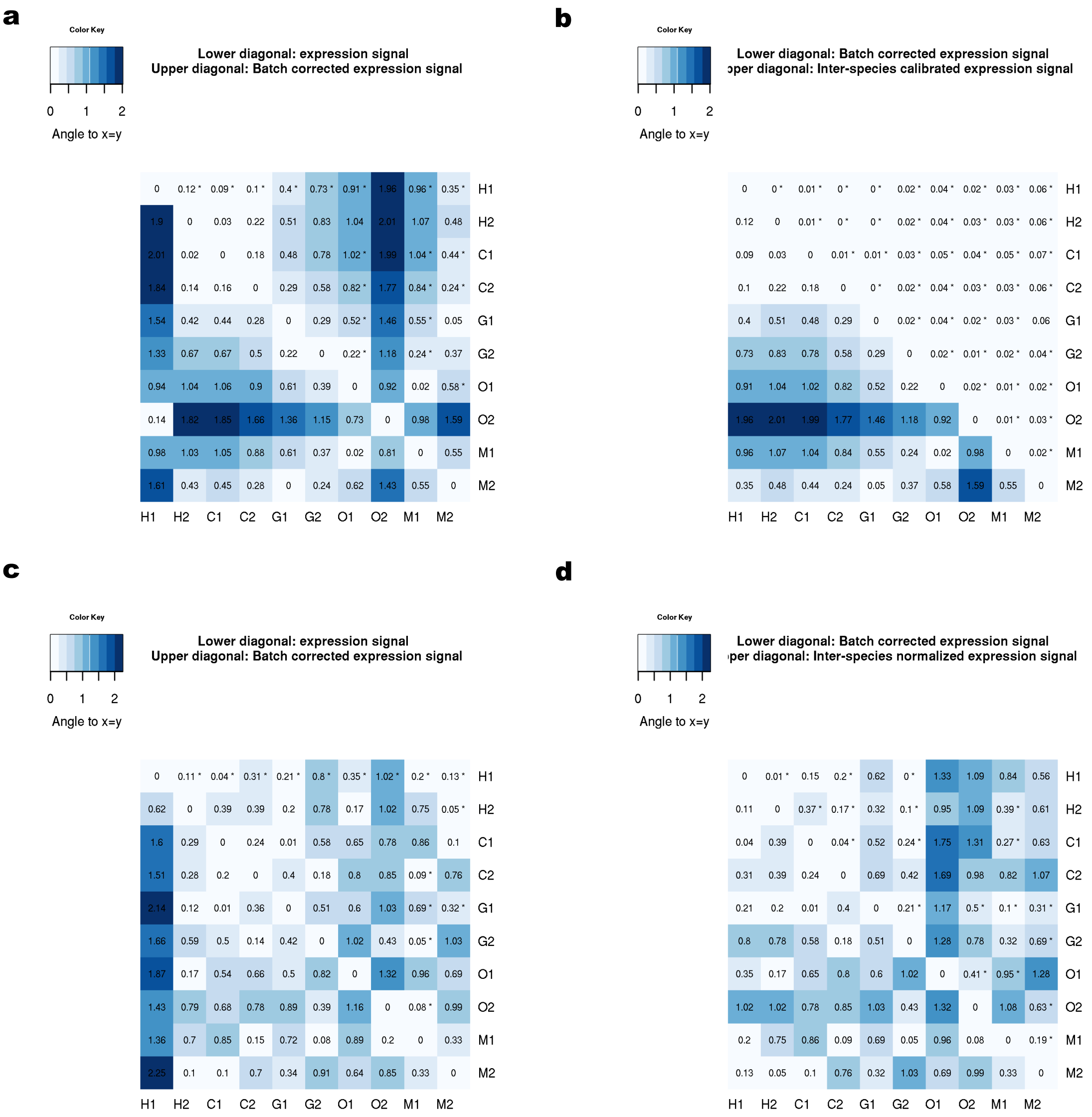

Supplementary Figure 39. Our signal calibration method effectively reduces noise variability. Cell values correspond to the angle to identity line (Supplementary Methods, Supplementary Fig. 38) **a,** Batch correction effect on IRCs; **b,** Inter-species calibration effect on IRCs; **c,** Batch correction effect on 1-to-1 orthologous protein-coding genes excluding IRCs and **d,** Inter-species calibration effect on IRCs 1-to-1 orthologous protein-coding genes excluding IRCa. **a** and **c,** Lower diagonal cell values correspond to the original angles prior batch correction (α_1), whereas upper diagonal ones correspond to the angle after batch correction (α_2). **b** and **d,** Lower diagonal cell values correspond to the angles after batch correction (α_2), whereas upper diagonal cell values correspond to the angles after inter-species signal calibration (α_3).

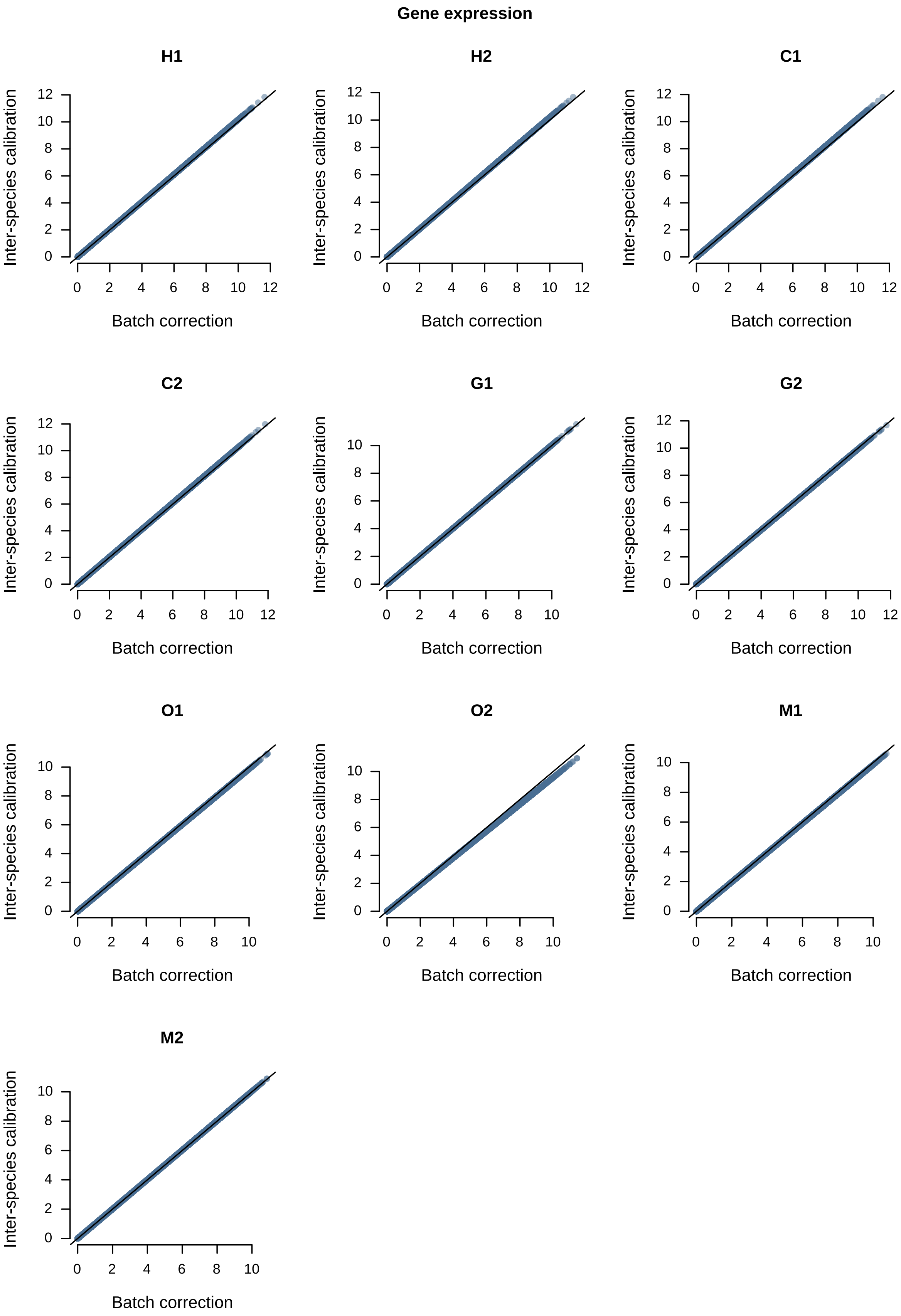

Supplementary Figure 40. Our signal calibration method effectively normalizes the signals at the tails of the distribution. Our signal calibration method outperforms quantile normalization (Supplementary Fig. 41) since it is able to handle properly the larger inter-

species differences at extreme values. For each sample included in this study, we plot the batch-corrected signal (x-axis) versus the calibrated signal (y-axis).

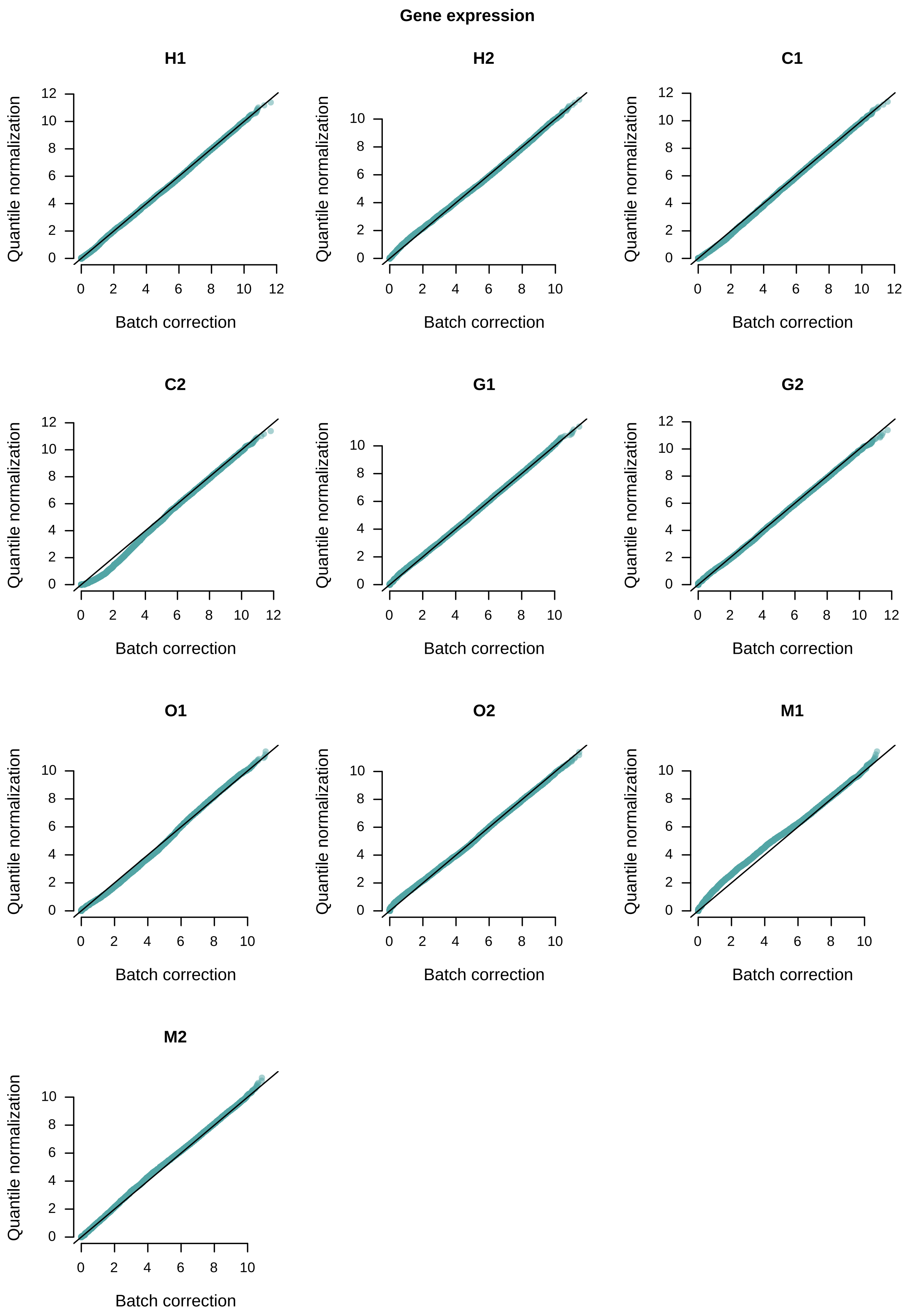

Supplementary Figure 41. Quantile normalization fails to properly normalize the signals at the tails of the distribution. For each sample included in this study, we plot the batch-corrected signal (x-axis) versus the quantile-normalized signal (y-axis). Note the deviation at the tails.

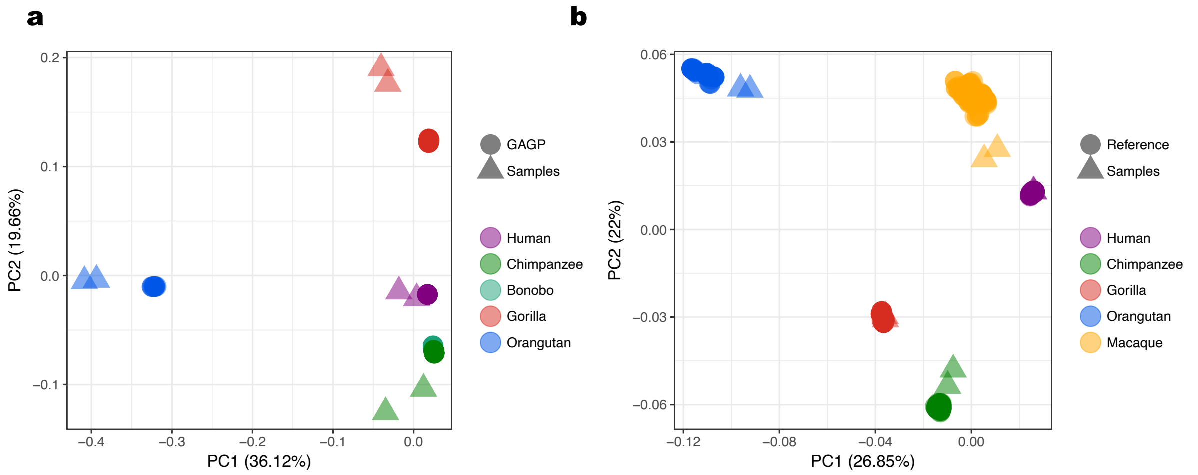

Supplementary Figure 42. Single nucleotide polymorphisms (SNPs) patterns recapitulate the known phylogenetic relationships between species. PCA based on **a,** autosomal (chr21) or **b,** mitochondrial SNPs. Triangles represent samples characterized in this study. Circles, samples from different studies (Supplementary Methods).

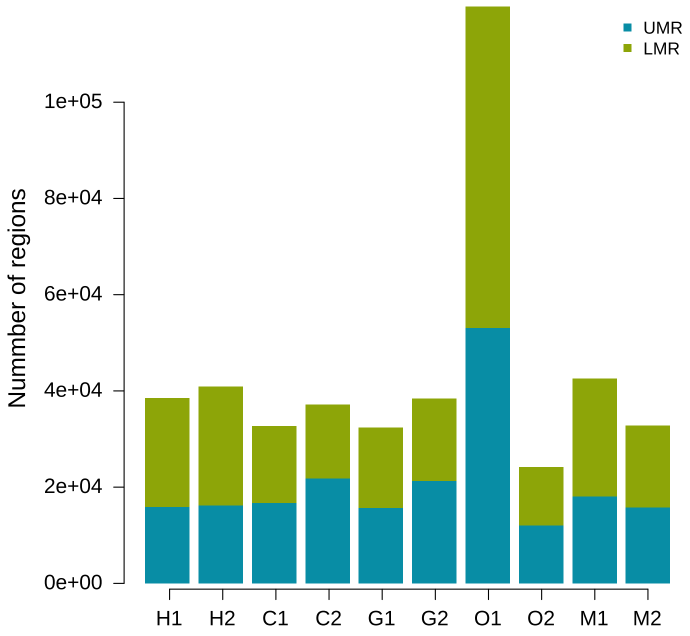

Supplementary Figure 43. Orangutan sample O1 has a disproportionate number of low methylated regions. Number of UMRs (unmethylated regions) and LMRs (low methylated regions) annotated per sample (Supplementary Methods).

Supplementary Tables

Supplementary Table 1. Number of regulatory elements with promoter and enhancers states annotated in each species.

	Human	Chimpanzee	Gorilla	Orangutan	Macaque
**sP**	7,444	7,677	8,708	8,400	7,278
**pP**	1,103	1,156	787	790	930
**wP**	662	2,415	2,637	2,115	698
**aP**	885	941	1,153	1,414	839
**sE**	21,267	29,567	25,179	20,246	29,193
**pE**	3,078	3,518	1,719	2,313	4,892
**wE**	41,549	31,676	34,744	24,660	41,083
**aE**	18,833	9,883	12,530	15,612	11,786

Supplementary Table 2. Number of regulatory elements classified as each type of regulatory component and number of orphan regulatory elements per species.

	Human	Chimpanzee	Gorilla	Orangutan	Macaque
**gP**	14,191	10,932	10,171	9,399	11,365
**gE**	32,662	29,654	26,772	18,869	35,519
**prE**	6,409	5,289	5,866	3,693	6,767
**PiE**	6,190	6,635	5,188	4,125	4,548
**EiE**	1,437	1,969	1,733	1,033	1,208
**Orphan**	17,662	25,204	28,747	29,021	28,427

Supplementary Table 3. Number of expressed genes per sample.

Sample	Expressed genes (TPM>0.5)
**H1**	18,011
**H2**	19,662
**C1**	14,525
**C2**	14,557
**G1**	14,263
**G2**	13,308
**O1**	14,063
**O2**	13,308
**M1**	13,852
**M2**	14,177

Supplementary Table 4. Spearman's rank correlation ρ values between TPM expression levels in biological replicates in autosomal protein-coding genes.

Species	Spearman's rho
**Human**	0.96
**Chimpanzee**	0.97
**Gorilla**	0.96
**Orangutan**	0.92
**Macaque**	0.97

Supplementary Table 5. Species pairwise Spearman's rank correlation ρ values in 1-to-1 orthologous protein-coding genes.

	Human	Chimpanzee	Gorilla	Orangutan	Macaque
**Human**	1	0.92	0.9	0.87	0.86
**Chimpanzee**	0.92	1	0.92	0.88	0.87
**Gorilla**	0.9	0.92	1	0.89	0.87
**Orangutan**	0.87	0.88	0.89	1	0.84
**Macaque**	0.86	0.87	0.87	0.84	1

Supplementary Table 6. Number of orthologous regulatory regions with each type of regulatory state.

	Human	Chimpanzee	Gorilla	Orangutan	Macaque
**sP**	2,079	2,061	2,201	2,133	1,996
**pP**	173	159	103	95	182
**wP**	72	209	324	155	73
**aP**	187	152	208	228	159
**sE**	4,905	7,193	6,172	4,919	6,229
**pE**	729	742	391	561	1,054
**wE**	9,879	10,040	11,685	9,360	10,166
**aE**	5,369	3,338	3,549	5,204	3,571
**Non-RE**	5,310	4,809	4,070	6,048	5,273

Supplementary Table 7. Evolutionary conservation of promoters and enhancer states with different activity levels per species in orthologous regulatory regions. Evolutionary conservation is defined as the average number of species in which a particular epigenetic state is conserved.

	Human	Chimpanzee	Gorilla	Orangutan	Macaque
**sP**	4.82	4.86	4.74	4.81	4.86
**pP**	2.9	2.86	2.51	2.84	2.26
**wP**	3.07	1.8	1.7	2.1	2
**sE**	4.31	4.1	4.09	4.3	4.01
**pE**	4.06	4.04	4.68	4.26	3.55
**wE**	3.68	3.88	3.66	3.8	3.51

Supplementary Table 8. Gene expression variability explained by a generalized linear model of gene expression based on H3K27ac, H3K27me3 and H3K36me3 signals at genic promoters with promoter or enhancer states and intragenic enhancers with enhancer states and their interactions (15 variables).

Histone:Regulatory_component:Epigenetic_State	% explained variance
**H3K27ac_P_epiP**	27.16
**H3K27ac_gE_epiE**	6.76
**H3K27ac_P_epiE**	0.48
**H3K27me3_P_epiP**	1.97
**H3K27me3_gE_epiE**	10.35
**H3K27me3_P_epiE**	4.75
**H3K36me3_P_epiP**	1.44
**H3K36me3_gE_epiE**	5.1
**H3K36me3_P_epiE**	0.01
**H3K27ac_P_epiP:H3K27ac_gE_epiE**	6.52
**H3K27ac_gE_epiE:H3K27ac_P_epiE**	0.18
**H3K27me3_P_epiP:H3K27me3_gE_epiE**	0.57
**H3K27me3_gE_epiE:H3K27me3_P_epiE**	0.44
**H3K36me3_P_epiP:H3K36me3_gE_epiE**	1.02
**H3K36me3_gE_epiE:H3K36me3_P_epiE**	0.04

Supplementary Table 9. GTEx tissue group correspondence.

Tissue	Tissue Group
**Adipose - Visceral (Omentum)**	AdiposeTissue
**Adipose - Subcutaneous**	AdiposeTissue
**Adrenal Gland**	AdrenalGland
**Whole Blood**	Blood
**Artery - Coronary**	BloodVessel

**Artery - Aorta**	BloodVessel
**Artery - Tibial**	BloodVessel
**Brain - Substantia nigra**	Brain
**Brain - Spinal cord (cervical c-1)**	Brain
**Brain - Amygdala**	Brain
**Brain - Anterior cingulate cortex (BA24)**	Brain
**Brain - Hippocampus**	Brain
**Brain - Hypothalamus**	Brain
**Brain - Putamen (basal ganglia)**	Brain
**Brain - Cerebellar Hemisphere**	Brain
**Brain - Frontal Cortex (BA9)**	Brain
**Brain - Caudate (basal ganglia)**	Brain
**Brain - Nucleus accumbens (basal ganglia)**	Brain
**Brain - Cortex**	Brain
**Brain - Cerebellum**	Brain
**Breast - Mammary Tissue**	Breast
**Colon - Sigmoid**	Colon
**Colon - Transverse**	Colon
**Esophagus - Gastroesophageal Junction**	Esophagus
**Esophagus - Muscularis**	Esophagus
**Esophagus - Mucosa**	Esophagus
**Heart - Atrial Appendage**	Heart
**Heart - Left Ventricle**	Heart
**Kidney - Cortex**	Kidney
**Liver**	Liver
**Lung**	Lung
**Muscle - Skeletal**	Muscle
**Nerve - Tibial**	Nerve
**Ovary**	Ovary
**Pancreas**	Pancreas
**Pituitary**	Pituitary
**Prostate**	Prostate
**Minor Salivary Gland**	SalivaryGland
**Skin - Not Sun Exposed (Suprapubic)**	Skin
**Skin - Sun Exposed (Lower leg)**	Skin
**Small Intestine - Terminal Ileum**	SmallIntestine
**Spleen**	Spleen
**Stomach**	Stomach
**Testis**	Testis
**Thyroid**	Thyroid
**Uterus**	Uterus
**Vagina**	Vagina
**LCLs**	LCLs
**Fibroblasts**	Fibroblasts

Supplementary References

1. Andrews, S. FastQC: A quality control tool for high throughput sequence data. *Bioinformatics* (2010).
2. Martin, M. Cutadapt removes adapter sequences from high-throughput sequencing reads. *EMBnet.journal* vol. 17 10 (2011).
3. Marco-Sola, S., Sammeth, M., Guigó, R. & Ribeca, P. The GEM mapper: fast, accurate and versatile alignment by filtration. *Nature Methods* vol. 9 1185–1188 (2012).
4. Langmead, B. & Salzberg, S. L. Fast gapped-read alignment with Bowtie 2. *Nat. Methods* **9**, 357–359 (2012).
5. Li, H. A statistical framework for SNP calling, mutation discovery, association mapping and population genetical parameter estimation from sequencing data. *Bioinformatics* **27**, 2987–2993 (2011).
6. Zhang, Y. *et al.* Model-based analysis of ChIP-Seq (MACS). *Genome Biol.* **9**, R137 (2008).
7. Smith, J. H. & Edwards Deming, W. Statistical Adjustment of Data. *Journal of the American Statistical Association* vol. 40 380 (1945).
8. Pertea, M., Kim, D., Pertea, G. M., Leek, J. T. & Salzberg, S. L. Transcript-level expression analysis of RNA-seq experiments with HISAT, StringTie and Ballgown. *Nature Protocols* vol. 11 1650–1667 (2016).
9. Anders, S., Pyl, P. T. & Huber, W. HTSeq--a Python framework to work with high-throughput sequencing data. *Bioinformatics* **31**, 166–169 (2015).
10. Love, M. I., Huber, W. & Anders, S. Moderated estimation of fold change and dispersion for RNA-seq data with DESeq2. *Genome Biol.* **15**, 550 (2014).
11. Aken, B. L. *et al.* Ensembl 2017. *Nucleic Acids Res.* **45**, D635–D642 (2017).
12. Durinck, S., Spellman, P. T., Birney, E. & Huber, W. Mapping identifiers for the integration of genomic datasets with the R/Bioconductor package biomaRt. *Nature Protocols* vol. 4 1184–1191 (2009).
13. Kuhn, R. M., Haussler, D. & Kent, W. J. The UCSC genome browser and associated tools. *Brief.*

Bioinform. **14**, 144–161 (2013).

14. Quinlan, A. R. & Hall, I. M. BEDTools: a flexible suite of utilities for comparing genomic features. *Bioinformatics* **26**, 841–842 (2010).
15. Leek, J. T., Johnson, W. E., Parker, H. S., Jaffe, A. E. & Storey, J. D. The sva package for removing batch effects and other unwanted variation in high-throughput experiments. *Bioinformatics* **28**, 882–883 (2012).
16. Bolstad, B. M., Irizarry, R. A., Astrand, M. & Speed, T. P. A comparison of normalization methods for high density oligonucleotide array data based on variance and bias. *Bioinformatics* vol. 19 185–193 (2003).
17. Li, H. & Durbin, R. Fast and accurate short read alignment with Burrows-Wheeler transform. *Bioinformatics* **25**, 1754–1760 (2009).
18. McKenna, A. *et al.* The Genome Analysis Toolkit: a MapReduce framework for analyzing next-generation DNA sequencing data. *Genome Res.* **20**, 1297–1303 (2010).
19. Price, A. L. *et al.* Principal components analysis corrects for stratification in genome-wide association studies. *Nature Genetics* vol. 38 904–909 (2006).
20. de Manuel, M. *et al.* Chimpanzee genomic diversity reveals ancient admixture with bonobos. *Science* **354**, 477–481 (2016).
21. Koboldt, D. C. *et al.* VarScan 2: somatic mutation and copy number alteration discovery in cancer by exome sequencing. *Genome Res.* **22**, 568–576 (2012).
22. Willems, T. *et al.* Genome-wide profiling of heritable and de novo STR variations. *Nat. Methods* **14**, 590–592 (2017).
23. Krueger, F. & Andrews, S. R. Bismark: a flexible aligner and methylation caller for Bisulfite-Seq applications. *Bioinformatics* vol. 27 1571–1572 (2011).
24. Hansen, K. D. *et al.* Increased methylation variation in epigenetic domains across cancer types. *Nat. Genet.* **43**, 768–775 (2011).
25. Hansen, K. D., Langmead, B. & Irizarry, R. A. BSmooth: from whole genome bisulfite sequencing reads to differentially methylated regions. *Genome Biol.* **13**, R83 (2012).
26. Hansen, K. D. *et al.* Large-scale hypomethylated blocks associated with Epstein-Barr virus-

induced B-cell immortalization. *Genome Res.* **24**, 177–184 (2014).

27. Hernando-Herraez, I., Garcia-Perez, R., Sharp, A. J. & Marques-Bonet, T. DNA Methylation: Insights into Human Evolution. *PLoS Genet.* **11**, e1005661 (2015).
28. Burger, L., Gaidatzis, D., Schübeler, D. & Stadler, M. B. Identification of active regulatory regions from DNA methylation data. *Nucleic Acids Res.* **41**, e155 (2013).
29. Stadler, M. B. *et al.* DNA-binding factors shape the mouse methylome at distal regulatory regions. *Nature* **480**, 490–495 (2011).
