# Supplementary material for: Epigenomic profiling of primate lymphoblastoid cell lines reveals the evolutionary patterns of epigenetic activities in gene regulatory architectures
Source: Nat Commun. 2021 May 25;12:3116. doi: 10.1038/s41467-021-23397-1 (PMC8149829; doi:10.1038/s41467-021-23397-1)
Supplement: Supplementary file 2 — Description of Additional Supplementary Files [file 41467_2021_23397_MOESM2_ESM.pdf]

Description of Additional Supplementary Files

File name: Supplementary Data 1.
Description: Annotated regulatory elements for human, chimpanzee, gorilla, orangutan and macaque. Excel files (one excel sheet per species) include the genomic coordinates of the regulatory elements, the assigned epigenetic state in each replicate as well as at the species level, the type of regulatory components and the associated genes.

File name: Supplementary Data 2.
Description: Gene expression levels (TPM) for human, chimpanzee, gorilla, orangutan and macaque. Excel files include the Ensembl gene ID, the gene biotype and the expression level in each replicate.

File name: Supplementary Data 3.
Description Species regulatory state at each orthologous regulatory region, including the type of component assigned to the orthologous regulatory region and the associated gene(s).

File name: Supplementary Data 4.
Description Genomic coordinates of orthologous regulatory regions.

File name: Supplementary Data 5.
Description Sparse Partial Correlation Networks for the eigencomponents. Excel files include the partial correlation values and the corresponding P-values for the Sparse Partial Correlation Analyses performed for Gene expression and the eigencomponents. Partial correlations and P-values for analyses performed using all 1-to-1 orthologous protein coding genes associated with at least one regulatory element in all species (genes with defined gene regulatory architectures) and with the subset of genes with full regulatory architectures (associated with at least one regulatory element in every type of regulatory component).

File name: Supplementary Data 6.
Description Sparse Partial Correlation Networks for the residuals of the eigencomponents for all histone marks. Excel files include the partial correlation values and the corresponding P-values the Sparse Partial Correlation Analyses performed for Gene expression and the residuals of the eigencomponents for H3K4me1, H3K4me3, H3K27ac, H3K27me3 and H3K36me3 together. Partial correlations and P-values for analyses performed using all the genes with regulatory architectures and using genes with a full regulatory architecture are provided.

File name: Supplementary Data 7.
Description Sparse Partial Correlation Networks for all histone marks. Excel files include the partial correlation values and the corresponding P-values for the Sparse Partial Correlation Analyses performed for gene expression, H3K4me1, H3K4me3, H3K27ac, H3K27me3 and H3K36me3 together. Partial correlations and P-values for analyses performed using all 1-to-1 orthologous protein coding genes associated with at least one regulatory element in all species (genes with defined gene

regulatory architectures) and with the subset of genes with full regulatory architectures (associated with at least one regulatory element in every type of regulatory component).

File name: Supplementary Data 8.
Description Sparse Partial Correlation Networks for histone marks. Excel files, one per histone mark, include the partial correlation values and the corresponding P-values for each of the Sparse Partial Correlation Analysis performed for gene expression and H3K4me1, H3K4me3, H3K27ac, H3K27me3 and H3K36me3. Partial correlations and P-values are included for analyses performed using all 1-to-1 orthologous protein coding genes associated with at least one regulatory element in all species (genes with defined gene regulatory architectures) and with the subset of genes with full regulatory architectures (associated with at least one regulatory element in every type of regulatory component).

File name: Supplementary Data 9.
Description Gene expression variability explained by a generalized linear model of gene expression based on H3K4me3, H3K4me1, H3K27ac, H3K27me3 and H3K36me3 signals at all types of regulatory components, including both promoter and enhancer states for each component as well as all possible interaction terms (1,225 variables). Excel file.

File name: Supplementary Data 10.
Description GO term clustering with group labels used for representation.

File name: Supplementary Data 11.
Description Functional enrichment of genes associated with genic promoters (gP) with conserved strong promoter states (sP) compared to genes with associated genic promoters.

File name: Supplementary Data 12.
Description Functional enrichment of genes associated with intragenic enhancers (gE) with conserved strong enhancer states (sE) compared to genes with associated intragenic enhancers.

File name: Supplementary Data 13.
Description Functional enrichment of genes associated with proximal enhancers (prE) with conserved poised enhancer states (pE) compared to genes with associated proximal enhancers.

File name: Supplementary Data 14. Functional enrichment of genes associated with genic promoters (gP) with conserved poised enhancer states (pE) compared to genes with associated genic promoters.

File name: Supplementary Data 15.
Description Functional enrichment of genes associated with intragenic enhancers (gE) with conserved weak enhancer states (wE) compared to genes with associated intragenic enhancers.

File name: Supplementary Data 16.
Description Functional enrichment of genes associated with human-specific intragenic enhancers (gE) with weak enhancer states (wE) compared to genes associated with intragenic enhancers.

File name: Supplementary Data 17.

Description Effect sizes of significant pairwise comparisons (Wilcoxon-Nemenyi-McDonald-Thompson test; $P < 0.05$) of GTEx tissue median expression values within datasets of genes with conserved and human-specific regulatory elements.

File name: Supplementary Data 18.
Description Genomic coordinates of human-specific gains of intragenic enhancers with weak states and further characterization.

File name: Supplementary Data 19.
Description Genomic coordinates of open chromatin regions detected in each species. Excel files, one per species.

File name: Supplementary Data 20.
Description Mitochondrial reference sequences.

File name: Supplementary Data 21.
Description Genotyping statistics.

File name: Supplementary Data 22.
Description Genomic coordinates of unmethylated (UMR) and low methylated regions (LMR). Excel files, one per species.
